# IgLON5 autoimmune antibodies activate Tau via neuronal hyperactivity

**DOI:** 10.1126/sciadv.aec2042

**Published:** 2026-05-13

**Authors:** Bilge Askin, Cagla Kilic, César Cordero Gómez, Sophie Lan-Linh Duong, Alvaro Domingues-Baquero, Alexander Goihl, Karsten Nalbach, Joana Petushi, Pia Grundschöttel, Jessica Wagner, Valentine Thomas, Janne Lamberty, Emily Withers, Hanna Huber, Sabrina Huebschmann, Ekaterina Semenova, Paul Turko, Andrew G. Newman, Lisa Diez, Marc Beyer, Elena De Domenico, Peter Körtvelyessy, Dirk Reinhold, Anja Schneider, Jonas J. Neher, Thomas Ulas, Stefan F. Lichtenthaler, Benjamin R. Rost, Dietmar Schmitz, Harald Prüss, Susanne Wegmann

**Affiliations:** ^1^ German Center for Neurodegenerative Diseases (DZNE), Berlin, Germany.; ^2^ Einstein Center for Neurosciences (ECN), Charité – Universitätsmedizin Berlin, Berlin, Germany.; ^3^ Department of Neurology and Experimental Neurology, Charité – Universitätsmedizin Berlin, Berlin, Germany.; ^4^ Berlin Institute of Health (BIH) at Charité – Universitätsmedizin Berlin, Berlin, Germany.; ^5^ Institute of Molecular and Clinical Immunology, Otto-von-Guericke-University, Magdeburg, Germany.; ^6^ German Center for Neurodegenerative Diseases (DZNE), Munich, Germany.; ^7^ Munich Cluster for Systems Neurology (SyNergy), Munich, Germany.; ^8^ Neuroproteomics, School of Medicine and Health, Klinikum Rechts der Isar, Technical University of Munich, Munich, Germany.; ^9^ Platform for Single Cell Genomics and Epigenomics (PRECISE) at the German Center for Neurodegenerative Diseases and the University of Bonn, Bonn, Germany.; ^10^ Genomics and Immunoregulation, Life and Medical Sciences (LIMES) Institute, University of Bonn, Bonn, Germany.; ^11^ German Center for Neurodegenerative Diseases (DZNE), Bonn, Germany.; ^12^ Metabolic Biochemistry, Biomedical Center Munich (BMC), Faculty of Medicine, Ludwig Maximilian University, Munich, Germany.; ^13^ Institute of Integrative Neuroanatomy, Charité, Berlin, Germany.; ^14^ Institute of Cell Biology and Neurobiology, Charité – Universitätsmedizin Berlin, Berlin, Germany.; ^15^ German Center for Neurodegenerative Diseases (DZNE), Magdeburg, Germany.; ^16^ Neuroscience Research Center (NWFZ), Charité-Universitätsmedizin, Berlin, Germany.

## Abstract

Anti-IgLON5 disease is an autoimmune disease, in which autoantibodies (AABs) against the neuronal cell surface protein IgLON5 lead to profound brain dysfunction and Tau pathology. How α-IgLON5 AABs cause neuronal Tau protein pathology and neurodegeneration remains unclear. We find that patient-derived α-IgLON5 AABs cluster IgLON5 proteins with other cell surface proteins, leading to neuronal hyperactivity that triggers pathological Tau missorting and phosphorylation, typically observed early in Tau-related neurodegenerative diseases. In wild-type mice, α-IgLON5 AABs induce hippocampal Tau phosphorylation and neuroinflammatory responses. Our findings establish a causal link between the α-IgLON5 AABs and Tau pathology in anti-IgLON5 disease patients and highlight the role of neuronal hyperactivity as a disease-overarching driver of Tau pathology and provide a potential target for therapeutic intervention.

## Teaser

α-IgLON5 autoantibodies induce clustering of neuronal cell surface proteins, acute neuronal hyperactivity, and Tau missorting.

## INTRODUCTION

In autoimmune encephalitis disorders, autoimmune antibodies [autoantibodies (AABs)] directed against neuronal or glial epitopes in the brain can cause or promote neuroinflammation and neurodegeneration (*1*, *2*). Anti-IgLON5 disease is a rare but severe neurological disorder characterized by the presence of AABs targeting the neuronal cell surface protein IgLON5 (*3*). Individuals diagnosed with anti-IgLON5 disease demonstrate diverse clinical manifestations, commonly experiencing sleep behavior abnormalities, movement disorders, memory deficits, and seizures (*4*–*6*). Brains of anti-IgLON5 patients show intraneuronal accumulations of phosphorylated Tau protein in several regions, including brain stem, hypothalamus, cerebellum, hippocampus, and basal ganglia, as well as in the spinal cord (*7*, *8*). Similar neuronal Tau accumulation and aggregation are well-known as pathological hallmarks in primary and secondary tauopathies, including frontotemporal dementia (FTD) variants and Alzheimer’s disease (AD) (*9*, *10*). However, anti-IgLON5 disease differs from other tauopathies because of its unique autoimmune context, anatomical distribution of Tau pathology, and set of clinical manifestations (*3*). The occurrence of Tau pathology in anti-IgLON5 disease poses the fundamental questions of how AABs against a cell surface protein can trigger pathophysiological intracellular Tau changes.

IgLON5 is primarily expressed in neurons in the brain stem, medulla oblongata, thalamus, cerebral cortex, and cerebellum and, to a lesser degree, in other brain areas like the hippocampal formation and amygdala (*11*). It belongs to the IgLON protein family, a group of immunoglobulin (Ig) domain cell adhesion molecules comprising five members [OPCML (=IgLON1), NTM (=IgLON2), LSAMP (=IgLON3), NEGR1 (=IgLON4), and IgLON5] (*12*). IgLON5 is a glycosylphosphatidylinositol (GPI)–anchored surface protein with three Ig domains (Ig1, Ig2, and Ig3) that extend into the extracellular space and mediate homo- and heterodimer formation with other IgLON family members on the cell surface (*13*–*15*). The physiological function of IgLON5 is not fully understood, however, other IgLON family members have been implicated in neuronal cell adhesion, neurite growth, neural circuit, and synapse formation (*16*–*18*). In neuronal cultures, binding of AABs from anti-IgLON5 patient serum to surface IgLON5 was shown to induce protein-AAB complex internalization (*19*), and long-term treatment (up to 4 weeks) induced common neurodegenerative phenotypes like cytoskeleton disruption (*20*), synapse loss, and cell death (*21*).

Here, using purified α-IgLON5 AAB from anti-IgLON5 disease patient plasma, we show that α-IgLON5 AABs promote propathological Tau changes and neurotoxicity by inducing acute neuronal hyperactivity via surface clustering of ion channels, receptors, and adhesion proteins. The present findings establish an important novel molecular and cellular mechanism underlying Tau pathology in anti-IgLON5 disease, which, in turn, provides a foundation for the development of treatment strategies aimed at preventing Tau changes in autoimmune encephalitis patients.

## RESULTS

### α-IgLON5 AABs isolated from patient plasma

**Fig. 1.**
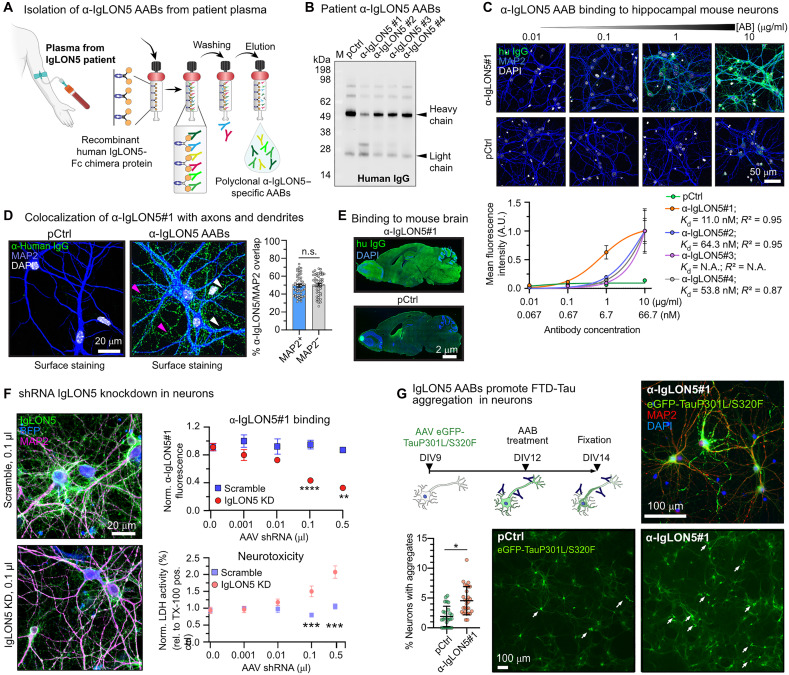
α-IgLON5 AABs from patient plasma bind neuronal IgLON5. (**A**) Principle of α-IgLON5 AAB isolation from clinically verified anti-IgLON5 patient plasma using affinity purification on immobilized recombinant human IgLON5-Fc chimera. (**B**) Western blot of patient α-IgLON5 AABs (IgLON5#1-4) and pCtrl probed with α-human IgG shows the presence of IgG heavy and light chains. (**C**) Dose-dependent binding of α-IgLON5 AABs and pCtrl to paraformaldehyde (PFA)–fixed primary hippocampal mouse neurons. *K*_d_’s were calculated via nonlinear fitting. [AB], antibody concentration. (**D**) Binding of α-IgLON5#1 to dendrites (MAP2^+^; white arrowheads) and axons (MAP2^−^; pink arrowheads). *n* = 3 experiments with three to five repeats per condition. Data points represent individual images analyzed. Student’s *t* test. n.s., not significant. (**E**) Immunoreactivity of α-IgLON5#1 and pCtrl in fresh-frozen, sagittal brain sections of an adult wild-type mouse. A.U., arbitrary units; N.A., not applicable. (**F**) Short hairpin RNA (shRNA)–mediated IgLON5 KD in primary neurons. Quantification of α-IgLON5#1 binding to neurons transduced with serial dilutions of AAVs encoding anti-mIgLON5 or scrambled shRNA. mIgLON5 shRNA AAV dose-dependent neurotoxicity [lactose dehydrogenase (LDH) assay] occurs ≧0.1 μl of shRNA AAVs. AAB signal was measured from dendrites (MAP2^+^ area). *n* = 3 experiments with three to five replicates. One-way analysis of variance (ANOVA) with Tukey postrest. (**G**) Experimental setup, representative images, and quantification of NFT-like Tau aggregation in neurons expressing enhanced green fluorescent protein (eGFP)–TauP301L/S320F for 5 days and then treated with α-IgLON5#1 or pCtrl (1 μg/ml) for 2 days. White arrows indicate Tau NFTs neurons. *n* = 3 experimental replicates. Data points are individual images. Student’s *t* test. All panels: Data are shown as means ± SEM. Scale bars as indicated.

To investigate the effects of α-IgLON5 AABs on neurons and Tau, we isolated polyclonal pools of IgLON5-specific AABs (α-IgLON5 AABs) from the plasma of four anti-IgLON5 disease patients through affinity chromatography with immobilized recombinant human IgLON5-Fc chimera (Fig. 1A). The purified α-IgLON5 AABs had a purity of 78 ± 6% [means ± SEM; signal intensity of heavy (~50 kDa) and light chain (~25 kDa) relative to total signal in SDS–polyacrylamide gel electrophoresis (PAGE); Fig. 1B]. Immunoblotting confirmed the binding of the isolated α-IgLON5 AAB pool to recombinant IgLON5 protein, but not to LGI1, another neuronal surface protein (fig. S1A), as well as the absence of residual IgLON5 protein in the AAB preparations (fig. S1B). The IgG subclass composition of isolated α-IgLON5 AAB pools [by enzyme-linked immunosorbent assay (ELISA)] appeared to be similar for all patient α-IgLON5 AAB preparations (fig. S1C), with 37 to 50% being IgG_1_, 37 to 52% being IgG_2_, 2 to 3% being IgG_3_, and 8 to 13% being IgG_4_. IgG pools derived from healthy subject serum (pCtrl) or commercially available human serum (IgG_pool_) showed similar proportion of IgG subclasses as well. Dot blot analysis of IgG subclasses confirmed the ELISA data for α-IgLON5#1 and pCtrl (fig. S1D). Additionally, we found that the IgM levels were comparable across samples, with a slight increase observed in α-IgLON5#4 and pCtrl (fig. S1E). pCtrl antibodies were subsequently used throughout the manuscript as control IgG pool.

### Patient-derived α-IgLON5 AABs bind neuronal cell surface IgLON5

To examine the surface antigen affinity of α-IgLON5 AABs, we measured their binding to the neuronal cell surface. Staining of paraformaldehyde (PFA)–fixed, unpermeabilized primary hippocampal mouse neurons [days in vitro (DIV)12] with α-IgLON5 AABs revealed different binding efficiencies, measured based on antihuman secondary antibody immunofluorescence (IF): α-IgLON5#1 [dissociation constant (*K*_d_) = 11 nM] showed the strongest and α-IgLON5#3 showed the weakest binding (Fig. 1C and fig. S1F). Further, we found that α-IgLON5 AABs (exemplified for α-IgLON5#1) bound similarly to cell bodies, dendrites (MAP2^+^), and axons (MAP2^−^) (Fig. 1D) and did not show a preference for presynaptic (synapsin-1) or postsynaptic (PSD95) areas (fig. S1G). We could also confirm reactivity of α-IgLON5#1 in mouse brain sections (Fig. 1E) and on the surface of cultured human neurons (fig. S1H). To confirm the specificity of α-IgLON5 AABs for IgLON5 protein, we confirmed dose-dependent binding of α-IgLON5#1 to human embryonic kidney (HEK) cells recombinantly expressing human IgLON5 protein (fig. S1I). Furthermore, in mouse neurons with short hairpin RNA (shRNA)–mediated IgLON5 knockdown (IgLON5 KD), the binding of α-IgLON5#1 decreased in a shRNA dose-dependent manner (Fig. 1F). IgLON5 KD in mouse neuroblastoma cells (Neuro2a) confirmed the efficiency of the IgLON5 shRNA approach (fig. S1J). Notably, the reduction of neuronal surface IgLON5 by ≥30% [at ≥0.1 μl of adeno-associated virus (AAV) shRNA] appeared to be neurotoxic (Fig. 1F), a finding consistent with previous reports (*19*). Last, to determine which part of IgLON5 protein was bound by α-IgLON5 AABs, we titrated α-IgLON5#1 to HEK cells recombinantly expressing human full-length IgLON5 or (combinations of) its individual GPI-anchored Ig domains (Ig1/2/3) (fig. S1K). These experiments showed binding of the polyclonal α-IgLON5#1 pool to all three Ig domains. Notably, previous studies suggested binding of nonpurified IgLON5 AABs in patient serum mostly to Ig2 (*19*).

### α-IgLON5 AABs induce Tau missorting

Most IgLON5 patients develop neuronal Tau accumulation and aggregation in one or more brain regions (*3*, *7*, *22*). To test whether α-IgLON5 AABs would be able to promote Tau aggregation, we applied α-IgLON5#1 on neurons that recombinantly expressed (AAV-mediated) human Tau FTD mutants with increased aggregation potential. In neurons expressing TauP301L or TauΔK280, α-IgLON5#1 treatment was unable to set off Tau aggregation (fig. S2A). However, in neurons expressing the spontaneously aggregating FTD–double-mutant TauP301L/S320F (*23*, *24*), α-IgLON5#1 treatment induced a significant increase in neurofibrillary tangle-like Tau aggregate formation compared with pCtrl-treated neurons (α-IgLON5#1, tangles in 5% of transduced neurons; pCtrl, tangles in 2% neurons; Fig. 1G). α-IgLON5 AABs, therefore, promoted spontaneous FTD-Tau aggregation.

**Fig. 2.**
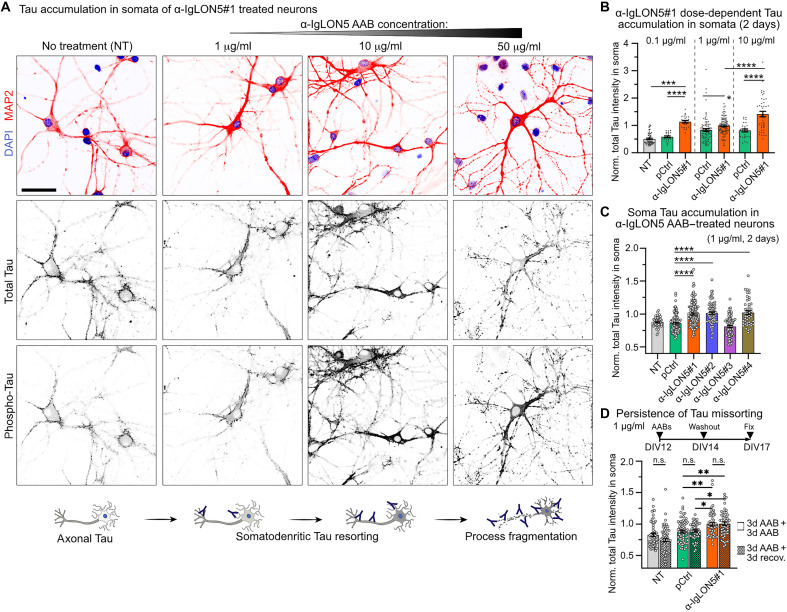
α-IgLON5 AABs promote somatodendritic Tau missorting. (**A**) Hippocampal neurons treated with increasing α-IgLON5#1 concentration (1, 10, and 50 μg/ml) for 2 days, immunolabeled for MAP2, total Tau, and phosphorylated Tau (pS199/pT205/pT231/pS396). Untreated neurons (NT) are shown for comparison. Treatment with α-IgLON5#1 (1 and 10 μg/ml) showed increased total Tau and phospho-Tau in neuronal cell bodies. At 50 μg/ml, fragmentation of neuronal processes indicates neurotoxicity and neurons showed strong phospho-Tau reactivity in cell bodies and elevated signal in the nucleus, whereas total Tau decreased in cell bodies. Scale bar, 50 μm. (**B**) Quantification of total Tau in neuronal cell bodies/somata treated with nontoxic doses of α-IgLON5#1 or pCtrl (0.1, 1, and 10 μg/ml) for 2 days. *n* = 3 experiments with three to five replicates. Data points show individual neurons (22 to 95 neurons per group). (**C**) Total Tau in cell bodies upon treatment with α-IgLON5 AABs (1 μg/ml) from four different patients or pCtrl for 2 days. *n* = 3 experiments with three to five replicates. Data points show individual neurons (43 to 136 neurons per group). (**D**) Total Tau in cell bodies upon treatment with α-IgLON5#1 or pCtrl (1 μg/ml) for 5 days continuously (bars without pattern fill) or for 2 days followed by 3 days (3d) without AABs (bars with pattern fill). *n* = 3 experiments with three to five replicates. Data points show individual neurons (48 to 71 neurons per group). [(B) to (D)] One-way ANOVA with Tukey posttest. n.s., not significant.

Missorting of phosphorylated Tau from the axon into the soma and dendrites is one of the earliest signs of Tau “activation” in the context of neuronal stress and neurodegenerative diseases like AD (*25*). Prolonged Tau missorting is thought to precede pathological Tau oligomerization and aggregation in AD and tauopathies (*26*). To assess whether α-IgLON5 AABs were able to induce a priori Tau missorting, we treated hippocampal neurons with the different α-IgLON5 AABs for 2 days and assessed the levels of endogenous Tau in neuronal somata by IF. Treatment with α-IgLON5#1 for 2 days induced somatic Tau accumulation in a dose-dependent manner (Fig. 2, A and B, and fig. S2B) and mild neurotoxicity over time (fig. S2C). Notably, overall Tau protein levels, as determined by pan-neuronal IF and Western blot of neuronal lysates, were unchanged by α-IgLON5 treatment, indicating no transcriptional or translational up-regulation of Tau (fig. S2, D and E). High α-IgLON5#1 concentration (50 μg/ml) induced fragmentation of neuronal processes already after 2 days (Fig. 2A), indicating specific or unspecific neurotoxicity. α-IgLON5#1, therefore, exerted a time- and dose-dependent toxicity. Similarly, prolonged treatment of cultured neurons with the total IgG fraction from α-IgLON5 patient serum, containing high doses of unpurified antibodies (e.g., AABs of 10 to 50 μg/ml for 5 to 21 days), was reported to induce neurotoxicity (*19*, *21*).

Tau accumulation in neuronal cell bodies also occurred upon treatment with AABs from other anti-IgLON5 patients, α-IgLON5#2 and α-IgLON5#4, that also showed strong binding to the neuronal surface (Fig. 1C), however, not for the weaker binding α-IgLON5#3 (Fig. 2C and fig. S2F). Together, these data suggested that binding of IgLON5 AABs can directly trigger Tau missorting. When removing α-IgLON5 AABs from the medium after 2 days and quantifying cell body Tau IF 3 days later, the cell body Tau levels remained similar to conditions of continuous treatment with AABs (Fig. 2D), suggesting that Tau, once resorted, resides in the somatodendritic compartment for multiple days. Notably, by Western blot, overall total and phosphorylated Tau (AT8, PHF-1, AT180, and pS199 epitopes) were similar in whole cell lysates of α-IgLON5 AAB–, pCtrl-, and nontreated (NT) neurons (fig. S2, G and H). This was likely due to generally high Tau phosphorylation in cultured neurons at baseline (*27*).

### α-IgLON5 AABs induce hippocampal Tau phosphorylation

**Fig. 3.**
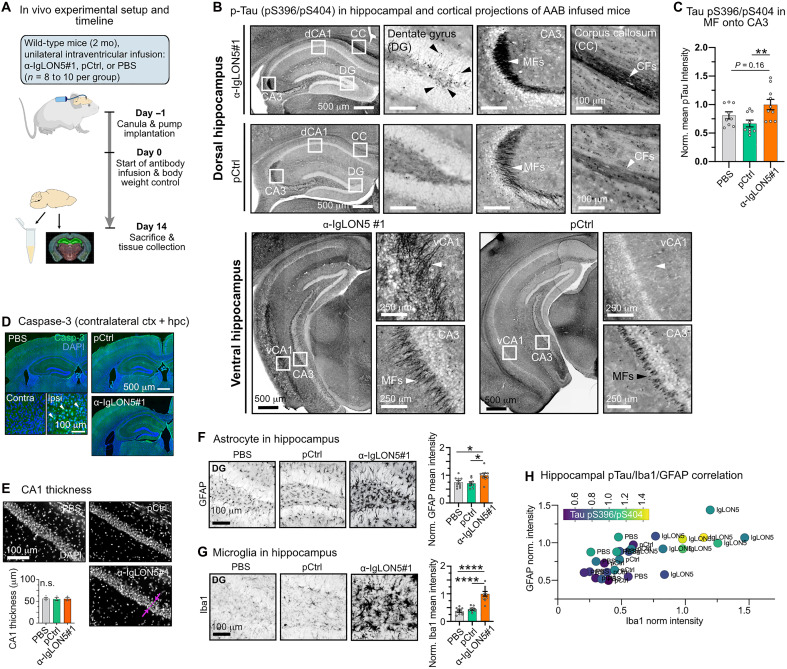
Intraventricular α-IgLON5 AAB infusion triggers hippocampal Tau phosphorylation and glia responses. (**A**) Experimental design for in vivo application of α-IgLON5 AABs. Wild-type mice were infused in the right ventricle with 75 μg of α-IgLON5#1 or pCtrl, or with PBS, over 14 days. At day 14, serum and brain tissue for protein and RNA analysis and IF were collected. 2 mo, 2 months. (**B**) p-Tau pS396/pS404 immunoreactivity in α-IgLON5#1 and pCtrl mouse brains. p-Tau accumulation occurs in dorsal hippocampi (top) in dentate gyrus (DG) granule cells (black arrowheads), mossy fiber (MF) projections onto CA3 (white arrowhead), and commissural fiber (CF) tracks of the corpus callosum (CC; white arrowhead). In ventral hippocampi, p-Tau accumulation is found in projections onto ventral CA1 (vCA1; white arrowhead) and MF projections onto CA3 (white arrowhead). (**C**) Quantification of p-Tau pS396/pS404 intensity in projections onto CA3. *n* = 8 to 10 animals per group, three to five brain sections per animal. (**D**) Immunolabeling of caspase-3 in α-IgLON5#1 and control brains. Caspase-3^+^ cells (white arrowheads) are only found near the injection site. (**E**) CA1 pyramidal layer in α-IgLON5#1 and control brains, with quantification of CA1 layer thickness (measured as indicated by pink line with arrowheads). *n* = 3 to 4 animals, three to four brain sections per animal. n.s., not significant. (**F**) Astrocytes [glial fibrillary acidic protein (GFAP)^+^] in DG of α-IgLON5#1 and control animals, with GFAP intensity quantification in entire hippocampal area. *n* = 8 to 10 animals, three to five brain sections per animal. (**G**) Microglia (Iba1) in DG of α-IgLON5#1 and control animals, with quantification of Iba1 intensity in entire hippocampal area. *n* = 8 to 10 animals, three to five brain sections per animal. (**H**) Triple correlation of hippocampal GFAP versus Iba and p-Tau pS396/pS404 (color gradient) fluorescence. Data points represent individual animals. [(C) and (E) to (G)] Data are shown as means ± SEM. One-way ANOVA with Tukey posttest. [(B) and (D) to (F)] Scale bars as indicated.

Next, we assessed whether α-IgLON5 AABs would be capable and sufficient of inducing Tau changes in vivo. For this, we delivered α-IgLON5 AABs into the brains of adult wild-type mice, which usually do not develop Tau aggregation pathology but can show abnormal Tau phosphorylation in sporadic tauopathy–associated conditions [e.g., seizures (*28*, *29*) or traumatic brain injury (*30*)]. Over the course of 14 days, a total amount of 75 μg (~3 μg/g body weight) of α-IgLON5#1 was infused via Alzet pumps into the right lateral ventricle (*n* = 10 animals). Littermates infused with the same amount and concentration of pCtrl (*n* = 9) or with phosphate-buffered saline (PBS; *n* = 9) functioned as controls. After the infusion, the brains were analyzed for phospho-Tau and neuroinflammation by immunohistology, and RNA and protein were extracted from the contralateral hemisphere for transcriptome and protein analyses (Fig. 3A).

Immunodetection of α-IgLON5#1 showed strong labeling in brain areas around the infused ventricle (cortex, hippocampus, and striatum), where the AAB concentration is expected to be the highest, and weaker labeling in more distant ipsilateral regions (e.g., parts of thalamus, hypothalamus, and amygdala) (fig. S3A). In the contralateral hemisphere, mostly, the hippocampus showed α-IgLON5#1 immunoreactivity. Animals treated with pCtrl, although having received the same antibody dose, showed generally less labeling, also in the contralateral hippocampus. This is in line with the weak, unspecific binding of pCtrl observed in neuronal cultures. In the following, we analyzed only tissue from contralateral hemispheres to avoid artifacts induced by injury and scar formation associated with the cannula implantation. Immunolabeling of brain sections revealed higher levels of phosphorylated Tau-pS396/pS404 in the contralateral hippocampus of α-IgLON5#1 compared with that of control (pCtrl and PBS) mice, particularly in mossy fiber (MF) projections onto CA3 (IgLON5#1 versus pCtrl, *P* = 0.01). Tau-pS396/pS404 could also be found in some cell bodies in dentate gyrus (DG), in bidirectional projections on ventral (but not dorsal) CA1, and in commissural fibers of the corpus callosum (CC; Fig. 3, B and C). In other brain regions (including spinal cord, fimbria, cerebellum, and hypothalamus but excluding brain stem and entorhinal cortex for sampling reasons), we did not observe Tau-pS396/pS404 immunoreactivity. For Tau-pS202/pT205 (AT8 epitope) and Tau-pT231, two other epitopes phosphorylated by Tau kinases like GSK3β, CdK5, and c-Jun N-terminal kinase and commonly associated with Tau pathology in neurodegenerative diseases (*31*), we observed no enhanced immunoreactivity compared with that of pCtrl-injected animals (fig. S3B). Notably, detecting Tau changes in wild-type mice, normally rather resilient to pathological changes in endogenous mouse Tau, demonstrates the strong potential of α-IgLON5 AABs in triggering Tau changes. The occurrence of Tau phosphorylation in the hippocampus, and not in brain regions majorly affected in IgLON5 disease brains, i.e., brain stem, hypothalamus, and cerebellum, likely relates to differences in α-IgLON5 AAB distribution in the brains of ICV-infused mice versus patients (Supplementary Text).

Next, we examined whether the increased Tau phosphorylation induced by α-IgLON5 AAB infusion was associated with neurotoxicity. However, we found no signs of apoptosis in the hippocampus, assessed on the basis of the absence of caspase-3–positive cells (Fig. 3D) as well as immunolabeling of milk fat globule epidermal growth factor 8 [MFG-E8; (*32*)] (fig. S3C). Caspase-3–positive cells were only present in the ipsilateral cortex near the injection site due to tissue damage during pump implantation (Fig. 3D). We also did not observe neurodegeneration, assessed on the basis of CA1 neuronal layer thickness (Fig. 3E) and neurofilament light chain (Nfl) serum levels (fig. S3D) (*33*). Together, these data indicated that 2 weeks of cerebroventricular infusion of α-IgLON5 AABs induced local, hippocampal Tau phosphorylation in the absence of detectable neurotoxicity. However, α-IgLON5#1 animals had slightly lower body weight (nonsignificant) compared with control groups throughout the infusion period (fig. S3E).

### Neuroinflammation caused by α-IgLON5 AABs

Tau phosphorylation and missorting, before aggregation and neurodegeneration, were previously suggested to correlate with glia cell activation in tauopathy animal models and human brains (*34*, *35*). Similarly, we found that astrocytic glial fibrillary acidic protein (GFAP; Fig. 3F and fig. S3F) and microglial Iba1 intensities (Fig. 3G and fig. S3G) were significantly increased in the contralateral hippocampus of α-IgLON5#1–infused mice compared with that of pCtrl and PBS controls. On the animal level, hippocampal Iba1 and GFAP levels appeared to increase with MF Tau-pS396/pS404 intensity, based on linear regression (moderate correlation by spearman) (Fig. 3H and fig. S3H).

**Fig. 4.**
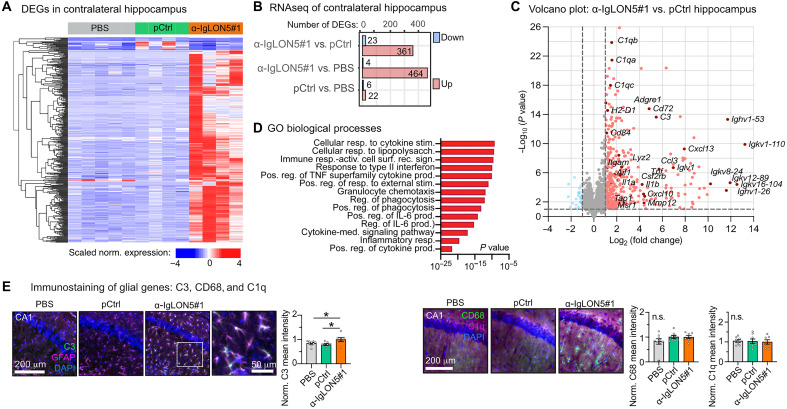
RNA-seq confirms microglia activation in α-IgLON5 AAB infused animals. (**A**) Hierarchical clustered heatmap of differentially expressed genes (DEGs) in contralateral hippocampi of PBS-, pCtrl-, and α-IgLON5#1–infused mice. (**B**) Number of significantly up-regulated (red) and down-regulated (blue) DEGs in contralateral hippocampi of PBS, pCtrl, and α-IgLON5#1 mice. (**C**) Volcano plot of gene deregulation in contralateral hippocampi of α-IgLON5#1 compared with those of pCtrl animals. (**D**) Gene Ontology (GO biological processes) enrichment analysis of up-regulated genes in hippocampi of α-IgLON5#1 compared with those of pCtrl animals. (**E**) Immunolabeling of neuroinflammatory markers (C3, CD68, and C1q) in hippocampi of α-IgLON5#1 and control mice. For the quantification of C3, CD68, and C1q fluorescence intensity, data are shown as means ± SEM; *n* = 7 to 9 animals, three to five brain sections per animal. Data are shown as means ± SEM. One-way ANOVA with Tukey posttest. Scale bars as indicated.

To gain further insights about α-IgLON5#1–induced effects in the brain, we analyzed the gene expression [by RNA sequencing (RNA-seq)] in contralateral hippocampi and cortices. In hippocampi of α-IgLON5#1, we detected 23 significantly down-regulated and 361 significantly up-regulated transcripts compared with those in pCtrl animals (Fig. 4, A and B, fig. S3I). Most of these transcripts were associated with inflammatory pathways [Gene Ontology (GO) biological processes] particularly interleukin-6 (IL-6) and tumor necrosis factor–α (TNFα) cytokine responses or belonged to Ig genes (Fig. 4, C and D, and data S1 and S2). The cortical transcriptome of the mice showed similar changes between α-IgLON5#1 and pCtrl animals, while only few differences in gene expression were found between the two control groups, pCtrl and PBS (fig. S3, J and K). Of the genes up-regulated in the hippocampus of α-IgLON5#1 animals, we confirmed the up-regulation of the neuroinflammation-associated astrocyte gene complement-3 (C3) by immunolabeling (Fig. 4E). Together, the in vivo data show that, in wild-type mice, cerebroventricular infusion of α-IgLON5 AABs induces localized Tau phosphorylation on epitope pS396/pS404 in distinct hippocampal axonal projections (MF and CC axons) as well as hippocampal neuroinflammation. Notably, because we observed neither phospho-Tau accumulation or aggregation in neuronal somata nor hippocampal neuronal loss, we interpret these changes as earliest, prepathological Tau changes that could lead to Tau pathology and neurotoxicity later on. Furthermore, in contrast to the animal model used here, in which mice received a direct AAB infusion selectively into the right ventricle for 2 weeks, anti-IgLON5 disease patients’ brains are diffusely exposed to the AAB over prolonged times. Such differences in AAB exposure likely explain the different distribution of Tau changes and neuroinflammation in humans, where Tau phosphorylation is mostly found in brain stem, hypothalamus, and cerebellum (*3*, *7*, *8*, *22*) (Supplementary Text).

### α-IgLON5 AABs trigger acute neuronal hyperactivity

Somatodendritic Tau missorting has previously been suggested to be driven by neuronal hyperactivity, for example, in the context of epileptic activity (*36*), Aβ oligomers (*37*), and chemical stimulation, e.g., glutamate (*37*). Because α-IgLON5 AABs induced Tau missorting, we hypothesized that α-IgLON5 AABs may exert neuronal hyperactivity.

**Fig. 5.**
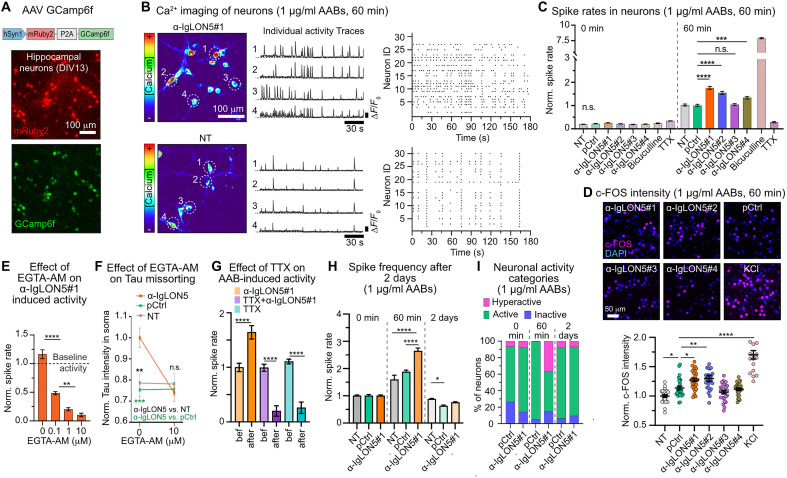
α-IgLON5 AABs induce neuronal hyperactivity. (**A**) Expression of mRuby2 and Ca^+2^ indicator GCamp6f in cultured neurons. (**B**) Ca^2+^ levels (time integrated; pseudocolored) in GCamp6f^+^ neurons treated with α-IgLON5#1 (1 μg/ml) or not (NT). White circles: cell bodies corresponding to GCamp6f traces (Δ*F*/*F*_0_ versus time). Raster plots: individual spikes of neurons in field of view (*n* = 25 to 30 cells). (**C**) Normalized spike rates after treatment with patient-derived α-IgLON5 AABs or pCtrl (1 μg/ml, 60 min). Spikes recorded before (0) and after (60 min) treatment in same cultures. Controls: bicuculline (30 μM, 5 min) and TTX (0.5 μM, 10 min). *n* = 3 experiments, 875 to 1344 neurons per group. Data are normalized to NT at 60 min. (**D**) c-FOS upon treatment with α-IgLON5 AABs or pCtrl (1 μg/ml, 60 min). Control: KCl (8 mM, 60 min). *n* = 3 experiments. (**E**) Spikes upon pretreatment with EGTA-AM concentrations for 20 min and then with α-IgLON5#1 (1 μg/ml, 60 min). Data are normalized to untreated neurons. *n* = 3 experiments, 203 to 309 neurons per condition. (**F**) Tau missorting in neurons pretreated or not with EGTA-AM (20 μM, 20 min) and then with α-IgLON5#1 or pCtrl (1 μg/ml, 2 days). *n* = 3 experiments, 47 to 60 neurons per condition. (**G**) Normalized spike rates before and after treatment with α-IgLON5#1 (1 μg/ml, 60 min), TTX (0.5 μM, 10 min), or both (first TTX and then α-IgLON5#1). *n* = 3 experiments. (**H**) Spikes before and after α-IgLON5#1 or pCtrl treatment (1 μg/ml, 60 min or 2 days). *n* = 3 to 4 experiments, 263 to 309 neurons per condition. (**I**) Percentage of inactive (blue), active (green), and hyperactive (pink) neurons upon treatment with α-IgLON5#1 or pCtrl (1 μg/ml for 0 min, 60 min, or 2 days). Hyperactivity: spikes/min > mean + 2*SD of pCtrl at each time point. Active: 0 < spikes/min < mean + 2*SD of pCtrl. Silent: no spikes. [(C) to (H)]: Means ± SEM, one-way ANOVA with Tukey posttest. [(A), (B), and (D)] Scale bars as indicated. n.s., not significant.

To test this hypothesis, we assessed spontaneous neuronal activity using calcium (Ca^2+^) imaging in GCamp6f-expressing neurons (Fig. 5A) treated with α-IgLON5 AABs or pCtrl (1 μg/ml). One hour after treatment, a significant increase in Ca^2+^ spike frequency could be detected for patient-derived AABs α-IgLON5#1, α-IgLON5#2, and α-IgLON5#4 compared with pCtrl or NT neurons (Fig. 5, B and C). α-IgLON5#3, which showed inefficient binding and did not induce Tau missorting (Figs. 1C and 2C), also did not increase neuronal activity. Activity-dependent expression of the immediate early gene c-FOS confirmed the observed increase in activity, showing significantly increased fluorescence intensity of nuclear c-FOS in α-IgLON5#1– and α-IgLON5#2–treated neurons (Fig. 5D). Even at a low AAB dose (0.1 μg/ml, ~0.67 nM), at which α-IgLON5#1 binding to neurons was similar to pCtrl (Fig. 1C), α-IgLON5#1 still induced a significant increase in neuronal activity compared with pCtrl (fig. S4, A to C). The mild activity induced by pCtrl in this condition can be accounted to the antibody application procedure, because a nonbinding human monoclonal antibody [mCtrl; (*38*)] showed a similar effect. In summary, these data indicated that α-IgLON5 AAB binding correlated with neuronal activity and Tau missorting (fig. S4, D and E). Application of EGTA-AM to suppress intracellular Ca^2+^ spikes (*39*) prevented somatodendritic Tau missorting (Fig. 5, E and F, and fig. S4F), and treatment of neurons with the sodium channels blocker tetrodotoxin (TTX) before α-IgLON5#1 treatment abolished α-IgLON5#1–induced Ca^2+^ transients and hyperactivity (Fig. 5G). These data establishing a causal link between α-IgLON5 AAB–induced hyperactivity increase in intracellular Ca^2+^ levels and Tau missorting and indicate that Ca^2+^ and sodium channels may be involved in α-IgLON5 AAB–induced activity.

Next, we investigated how α-IgLON5 AAB or pCtrl (1 μg/ml) exposure for 2 to 3 days would affect neuronal activity, a time point when Tau was missorted into neuronal cell bodies following α-IgLON5 AAB treatment. After the initial induction of neuronal hyperactivity within 60 min after α-IgLON5#1 application, both the average spike rate and the fraction of hyperactive neurons dropped back after 2 days to levels detected before treatment (Fig. 5, H and I, and fig. S4G). Baseline Ca^2+^ levels (*F*_0_ of gCam6 fluorescence) seemed to be not altered in actively spiking neurons but was reduced in silent neurons after 2 days of α-IgLON5#1 compared with pCtrl and NT neurons (fig. S4H), which could reflect a homeostatic reaction to prolonged hyperexcitation (*40*). The absence of hyperactivity after 2 days was confirmed by electrophysiological recordings in autaptic cultures of hippocampal neurons (fig. S4, I and J) treated with AABs for 3 days, which showed no difference between α-IgLON5#1 and pCtrl-treated cells for any measured parameters (fig. S4I). In vivo, activity-dependent c-FOS expression in the DG was similar in mice that had received α-IgLON5#1 or pCtrl antibodies for 2 weeks (fig. S4K). α-IgLON5 AAB–induced neuronal hyperactivity, therefore, seemed to be transient, whereas the related Tau missorting persisted over days.

Prolonged AAB exposure may lead to a reduction of synapse numbers, which may dampen the initial α-IgLON5 AAB–induced hyperexcitation. Previous data from neurons treated with high doses of antibodies from anti-IgLON5 disease patient serum (1:50 dilution of total IgG isolates) showed a loss of synaptic protein content indicative of synaptic decline (*21*). In our model of low-dose (1 μg/ml) α-IgLON5#1 treatment, however, no significant loss in pre- and postsynaptic marker densities (fig. S4, L and M) was observed compared with that in control groups up to 7 days of AAB treatment.

### α-IgLON5 AABs cluster cell adhesion and ion channel proteins

**Fig. 6.**
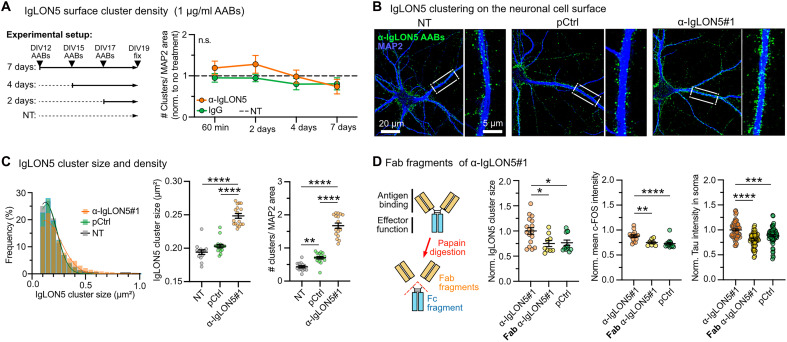
α-IgLON5 AAB–induced IgLON5 protein clustering on the cell surface. (**A**) Quantification of IgLON5 surface cluster density (# clusters/MAP^+^ area) in primary neurons treated with α-IgLON5#1 or pCtrl (1 μg/ml) at indicated time points (60 min, 2 days, 4 days, and 7 days). *n* = 3 experiments with three to four replicates per condition. Two-way ANOVA with Šidák posttest. (**B**) Confocal images of IgLON5 surface clusters on nonpermeabilized neurons upon treatment with α-IgLON5#1 or pCtrl (1 μg/ml) for 60 min. Scale bars as indicated. (**C**) Refined analysis of IgLON5 surface cluster density and size. Histogram shows frequency distribution of IgLON5 surface cluster size for all measured clusters. Additionally, mean cluster size and density (#IgLON5 clusters/MAP^+^ area) are shown. *n* = 3 experiments, three replicates per condition. One-way ANOVA with Tukey posttest. (**D**) α-IgLON5#1 and pCtrl Fab fragment (antigen-binding region) generation by papain digestion. IgLON5 surface cluster size/area, neuronal activity (nuclear c-FOS intensity), and Tau accumulation in cell bodies in upon treatment with equimolecular concentrations of α-IgLON5#1, Fab α-IgLON5#1, and pCtrl. For analysis of cluster size and neuronal activity, neurons were treated for 60 min. For Tau missorting, neurons were treated for 2 days. *n* = 3 experiments, four to five replicates per condition. One-way ANOVA with Tukey posttest. [(A) to (C)] Data are shown as means ± SEM.

The decline of hyperactivity after multiple days of α-IgLON5 AAB treatment could be due to internalization of IgLON5 protein/antibody complexes, leading to a decrease in IgLON5 cell surface epitopes (*2*, *19*, *41*). Previous studies reported an irreversible internalization of surface IgLON5 clusters in neurons after 3 days of treatment with the bulk antibody fraction from α-IgLON5 patient serum (*19*). In neurons continuously treated with α-IgLON5#1 over multiple days, we found an initial increase in IgLON5 surface clusters compared with that in controls after 60 min, followed by a decline of IgLON5 clusters back to densities comparable to untreated control neurons after 7 days (Fig. 6A). To assess the intracellular fate of IgLON5 clusters on longer time scales, we conjugated α-IgLON5#1 to the pH-sensitive fluorophore pHrodo, which increases fluorescence upon exposure to acidic environments characteristic of late endosomes and lysosomes (pH ~4.5 to 5.5). pHrodo fluorescence was detected in neurons treated with α-IgLON5#1 for 2 days and, to a lesser extent, also in pCtrl-treated neurons (fig. S4N), which may be due to unspecific antibody/protein internalization by neurons. These data suggest that, at least, a fraction of internalized α-IgLON5#1 is trafficked to degradative compartments rather than being recycled to the plasma membrane. IgLON5 cluster internalization could, therefore, in part, account for the observed reduction in hyperactivity after 2 to 3 days.

AAB-induced clustering of surface receptors, i.e., *N*-methyl-d-aspartate receptors (NMDARs) and LGI1, can change receptor surface availability and dynamics, leading to altered neuronal activity (*42*–*48*). We, therefore, hypothesized that AAB-induced IgLON5 clustering may be involved in neuronal hyperactivity as well. Untreated cultured neurons showed a clustered appearance of IgLON5 on the cell surface [Fig 5B(*19*)]. Acute (60 min) treatment with α-IgLON5 AABs up-regulated IgLON5 surface cluster size and their dendritic density (Fig. 6, A to C), implicating antibody-mediated redistribution of IgLON5 molecules into larger surface clusters. Notably, total IgLON5 surface fluorescence was slightly reduced (fig. S5A), with ~7.5% of the IgLON5 IF signal detected intracellularly (fig. S4O), suggesting that a small fraction of IgLON5 clusters were internalized within the first hour of treatment. This occurred at maintained total cell surface labeling of IgLON5, indicating no increase in IgLON5 protein content (fig. S5A)

To test whether α-IgLON5 AABs directly induced the clustering, we separated the antigen-recognizing Fab fragments from the connecting Fc part of α-IgLON5#1 by papain digestion (fig. S5B), which removes the ability of the AAB to bind and “cross-link” multiple IgLON5 molecules at a time. This approach was previously used to disable clustering of NMDARs by α-NMDAR AABs (*49*). For α-IgLON5#1, treatment with equimolar concentrations of α-IgLON5#1 Fab fragments (2 mol of Fab fragments for 1 mol of IgG) did not induce IgLON5 surface clustering as observed for intact α-IgLON5#1 (Fig. 6D and fig. S5C). α-IgLON5#1 Fab fragments also induced less neuronal activity (c-FOS intensity) and Tau missorting (Fig. 6D). Hence, α-IgLON5#1–induced IgLON5 clustering seemed to be involved in inducing neuronal hyperactivity and Tau changes.

**Fig. 7.**
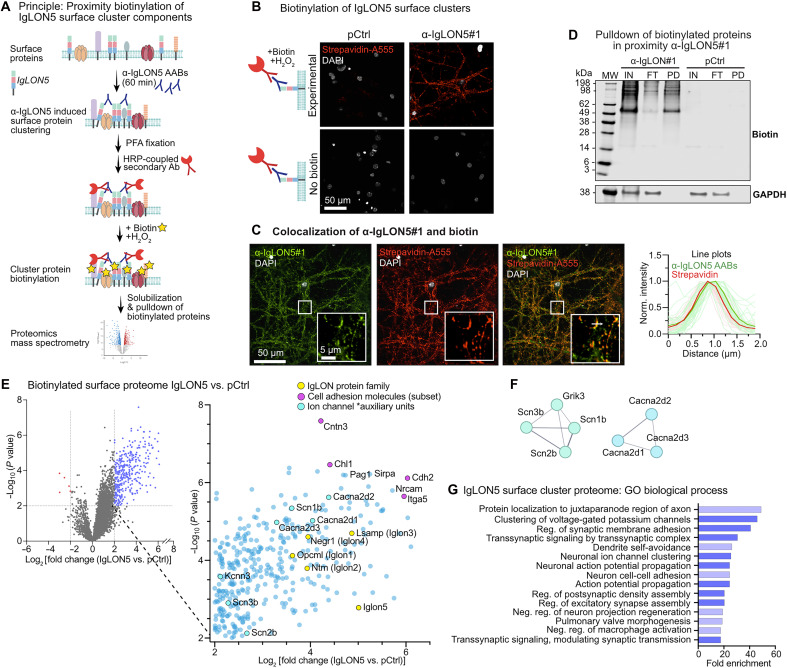
α-IgLON5 AAB–induced clustering of cell surface proteins. (**A**) Antibody-mediated proximity biotinylation assay. Live hippocampal neurons were treated with α-IgLON5#1 for 60 min to allow surface clustering, then fixed, and incubated with α-human IgG–horseradish peroxidase (HRP). Membrane-impermeant biotin-phenol and H_2_O_2_ were applied to selectively label surface proteins in proximity to AAB-bound IgLON5 clusters. Biotinylated proteins were pulled down and analyzed by MS. Surface protein biotinylation could be observed based on streptavidin–Alexa Fluor 555 labeling in neurons treated with α-IgLON5#1, α-human IgG-HRP, and biotin. Ab, antibody. (**B**) Detection of IgLON5 surface cluster biotinylation with streptavidin–Alexa Fluor 555 (A555) upon incubation of neurons with α-IgLON5#1 (1 μg/ml), followed by fixation, HRP secondary incubation, and biotinylation reaction. Scale bar as indicated. (**C**) Images of overlapping α-IgLON5 and streptavidin–Alexa Fluor 555 signal in neurons treated with α-IgLON5#1 (1 μg/ml) for 60 min. Intensity profile plot confirms colocalization of α-IgLON5 AABs and biotin signal. Thick lines indicate mean profiles, and thin lines indicate individual measured profiles. Scale bars as indicated. (**D**) Example Western blot showing biotinylated proteins in input (IN; cell lysate), flow-through (FT; wash), and pulldown (PD). Note: Glyceraldehyde-3-phosphate dehydrogenase (GAPDH) is only detected in IN and FT samples, confirming cell surface specificity of the labeling. (**E**) Volcano plot of biotinylated surface proteins in neurons treated with α-IgLON5#1 versus pCtrl. Proteins significantly enriched in IgLON5 clusters (blue points) are provided as magnified view: IgLON family members (yellow; IgLON51/2/3/4/5), cell adhesion proteins (pink), and ion channel subunits (cyan). (**F**) Interaction network (STRING data base) of selected ion channel auxiliary subunits found in IgLON5 clusters (from fig. S5E). (**G**) GO analysis of biological processes associated with proteins significantly enriched in α-IgLON5 AAB–induced surface clusters.

To determine whether IgLON5 clusters contained surface molecules capable of inducing neuronal hyperactivity, we determined the IgLON5 cluster proteome using an immunolabeling-based proximity biotinylation approach (Fig. 7, A and F, and fig. S5D). Neurons treated with α-IgLON5#1 or pCtrl for 60 min were fixed (4% PFA in PBS) and immunolabeled with α-human IgG secondary coupled to horseradish peroxidase (HRP). Addition of cell-impermeable biotin and hydrogen peroxide (H_2_O_2_) catalyzed the biotinylation of cell surface proteins in proximity to IgLON5#1, hence, in IgLON5 cell surface clusters. By microscopy, the spatial overlap and restriction of biotinylation to IgLON5 clusters could be confirmed (Fig. 7, B and C). We, therefore, considered biotinylated proteins in α-IgLON5#1–treated neurons to be part of IgLON5 clusters. Western blots confirmed the biotinylation of proteins in lysates of α-IgLON5#1–treated neurons and, to a lesser degree, in pCtrl-treated neurons (Fig. 7D) and the successful pulldown of these proteins using streptavidin beads. Proteomics mass spectrometry (MS) revealed the enrichment of specific cell surface proteins in IgLON5 clusters: other members of the IgLON family (IgLON1/2/3/4), as expected from a previous study (*15*), as well as other cell adhesion molecules [i.e., Ncam, Nrcam, Cadherins (Cdh4, Cdh6, Cdh11, and Cdh2), Contactins (Cntn1-6), and Integrins (Itga5, Itga7, and Itgb1); Fig. 7E and data S3]. IgLON5 clusters also contained ion channel auxiliary units involved in the regulation of neuronal activity, particularly regulatory subunits of voltage-gated sodium channels (Scn1b/2b/3b) and voltage-gated Ca^2+^ channels (Cacna2d1/d2/d3), voltage-gated potassium channel KCNN3, and the kainate receptor subunit GRIK3 (Fig. 7F and fig. S5E). Notably, we did not detect Na_v_ and Ca_v_ in the IgLON5 clusters proteome. This may be attributed to their embedding in the plasma membrane, which renders the channels difficult to extract from fixed neurons and may make them inaccessible for the biotinylation reaction using non–cell-permeable biotin. GO analysis (GO biological processes) of all differentially enriched proteins revealed proteins involved in cell adhesion, synapse assembly and function, synaptic and axonal signal transmission and propagation, and ion channel clustering (Fig. 7G, fig. S5F, and data S4). The proteome of the input cell lysate (before streptavidin bead pulldown) of α-IgLON5#1 and pCtrl-treated neurons did not have significant differences (fig. S5G and data S3).

In summary, our data suggest that the binding of α-IgLON5 AABs induces physical clustering and stimulation of Na_v_, Ca_v_, and K_v_ channels and auxiliary subunits and the glutamate-sensing kainate receptor subunit GRIK3. The deregulation of this complex mix of activity regulating channels and receptors by α-IgLON5 AABs explains the acute and excessive hyperexcitability of neurons upon α-IgLON5 AABs binding, which ultimately triggers prolonged somatodendritic Tau missorting and neurotoxicity. In addition, α-IgLON5 AABs induce clustering of cell adhesion molecules, including cadherins and integrins, a fundamental step at the top of cell adhesion signaling cascades. Whether cell adhesion signaling contributes to α-IgLON5 AAB–induced Tau changes has to be further investigated.

## DISCUSSION

AABs against neuronal receptors and cell surface proteins, e.g., NMDAR, GABAAR, and LGI1, can alter neuronal physiology (*1*, *2*, *50*–*52*). Our data reveal that anti-IgLON5 disease may involve a similar disease mechanism: Cell surface binding of α-IgLON5 AABs promotes the physical clustering of surface IgLON5 with cell adhesion molecules and ion channels, leading to acute neuronal hyperactivity that induces persistent Tau missorting, promotes Tau aggregation, and, ultimately, can lead to neurotoxicity. Previous studies reported that long-term treatment (for weeks) of neurons in vitro or of mice with anti-IgLON5 disease patient serum antibody pools (not enriched for α-IgLON5 AABs) disrupted synaptic protein levels and neuronal activity and caused cognitive deficits and anxiety-like behavior in mice (*21*, *53*, *54*). Our data now shed light on the events upstream of these effects.

Antibody-induced changes in receptor surface distribution have previously been reported for AABs. For example, AABs against LGI1 reduce its surface availability and impair interactions with potassium channels, leading to destabilization of synaptic contacts, dysregulation of excitatory transmission, and initiation of pathological hyperactivity including seizures (*45*, *46*, *48*, *52*, *55*). α-NMDAR AABs trigger nanoscale clustering of NMDARs, disrupting their association with scaffolding proteins, followed by receptor internalization and degradation, ultimately leading to a reduction in surface NMDARs and impaired synaptic transmission and neuronal silencing (*43*, *44*, *49*, *51*). For α-IgLON5 AABs, we similarly found that binding induces clustering of IgLON5 together with cell surface proteins. After 2 days, however, the enhanced IgLON5 receptor/protein clustering disappeared, and we detected IgLON5 antibodies within acidic late endosomal/lysosomal compartments, suggesting that a fraction of internalized complexes is routed toward degradative pathways rather than being rapidly recycled to the plasma membrane. Such irreversible internalization and lysosomal degradation of IgLON5 antibody–protein complexes is in line with previous reports (*19*). If α-IgLON5 AAB–induced hyperexcitation is directly related to IgLON5 clustering, as suggested by our data, then the reduced the availability of functional IgLON5 at the neuronal surface may limit the capacity of α-IgLON5 AABs to sustain receptor clustering and hyperexcitability, explaining why AAB-induced hyperexcitation vanished after 2 days in neuronal cultures. Notably, the shRNA-mediated reduction of IgLON5 led to a dose-dependent increase in neuronal toxicity, similar to what has previously been suggested for internalization-related reduced surface IgLON5 (*19*). Therefore, loss of surface IgLON5, whether through genetic manipulation or prolonged antibody-mediated internalization, appears to contribute to neuronal vulnerability.

α-IgLON5 AABs induce clustering of ion channel auxiliary units (Scn1b/2b/3b and Cacna2d1/d2/d3) that regulate the permeability of voltage-gated Ca^2+^ (Ca_v_) and sodium (Na_v_) channels, which are essential for synaptic transmission and action potential initiation and propagation (*56*), and with the Ca^2+^-activated potassium channel KCNN3 and the ionotropic glutamate receptor GRIK3/GLUR7, which are directly involved in neuronal excitability (*57*, *58*). Aberrant physical interactions and clustering of these proteins, induced by α-IgLON5 AABs, can have a profound impact on neuronal activity. In addition, we identified several cell adhesion molecules (IgLON family members, integrins, cadherins, protocadherins, and contactins) in IgLON5 surface clusters. These transmembrane proteins dynamically interact with the actin cytoskeleton to facilitate synaptic organization, neuronal migration, and development (*59*, *60*). Their interaction with other surface proteins modulates function and localization of voltage-gated ion channels, thereby altering Ca^2+^ influx, neuronal excitability, and other signaling pathways that remodel the cytoskeleton, strengthen adhesion, and support synaptic function and plasticity. The concerted effect of α-IgLON5 AAB binding on adhesion proteins, receptors, and ion channels can explain how α-IgLON5 AAB binding induces neuronal hyperexcitability. While these findings establish α-IgLON5 AAB–mediated protein clustering at the neuronal surface as a key upstream event driving hyperactivity and Tau activation, future studies using targeted pharmacological perturbations, e.g., using NMDA/AMPA receptor antagonists and Ca^2+^ channel blockers, will be required to identify specific molecular players in the cascade.

We showed that intracellular Ca^2+^ chelation can rescue α-IgLON5 AAB–induced Tau missorting. Whether the Ca^2+^ that activates Tau originates from synaptic influx and/or is released from internal stores, e.g., the endoplasmic reticulum (ER) or mitochondria, remains unclear. Previously, intracellular Ca^2+^ increase through NMDAR activation or membrane depolarization was shown to engage downstream pathways linked to Tau phosphorylation and truncation, including calpain activation, sustained extracellular signal–regulated kinase (ERK)/mitogen-activated protein kinase signaling and the CaMKK2-AMPK pathway (*61*–*63*). In addition, activity-dependent synaptic Ca^2+^ entry can trigger secondary Ca^2+^ release from intracellular stores, such as the ER, thereby amplifying and prolonging intracellular Ca^2+^ signaling (*64*), which could further promote and prolong Tau phosphorylation. For example, during nonhuman primate aging, cyclic adenosine 3′,5′-monophosphate (cAMP)–inducible cAMP-dependent protein kinase (PKA)–dependent phosphorylation of RyR2 at S2808 (pS2808-RyR2) causes Ca^2+^ leak from intracellular stores, correlating with Tau phosphorylation at CaMKII and GSK3β-dependent sites (*65*). Ultimately, identifying the contribution of synaptic Ca^2+^ influx and intracellular Ca^2+^ storage release, as well as the involved downstream Tau kinases, will be required to define the signaling cascades acting in Tau activation downstream of α-IgLON5 AAB binding at the neuronal surface.

In vitro, α-IgLON5 AABs triggered acute (minutes to hours) neuronal hyperactivity, which vanished after 2 days but left behind lasting (multiday) Tau missorting and promoted FTD-Tau aggregation. Similarly, in vivo, after 14 days of AAB infusion, we did not detect neuronal hippocampal activation using c-FOS in animals that received α-IgLON5 AABs but detected hippocampal Tau phosphorylation, potentially activity-induced. Whether α-IgLON5 AABs also promote FTD-Tau aggregation in mice remains to be tested. However, together, these findings may suggest a homeostatic adaptation of neuronal activity to initial α-IgLON5 AAB hyperexcitation, which is coupled to prolonged Tau phosphorylation and missorting. In anti-IgLON5 disease patients, where α-IgLON5 AABs are chronically present in the cerebrospinal fluid (CSF), and, therefore, neurons are repeatedly or continuously exposed to AABs over long time periods. Antibody-driven Tau changes and neuronal dysfunction may occur in episodes, modulated by fluctuations in local AAB concentration and state-dependent network activity (e.g., sleep-wake cycles). Over time, these recurrent “hits” could accumulate into gradual, irreversible, and neurotoxic accumulation of pathological Tau, contributing to anti-IgLON5 disease progression.

The extended presence of Tau in the somatodendritic compartment can exhaust neuronal homeostasis (*66*), induce synapse loss (*34*), and impair other key neuronal functions (*67*–*69*). Consequently, this would explain why immunotherapy (to deplete AABs) seems most beneficial when initiated early in anti-IgLON5 disease (*5*), i.e., before overt Tau accumulation. Tau pathology has previously been connected to neuronal hyperactivity in epilepsy (*70*) and in AD (*71*–*73*). Sustained neuronal hyperactivity, induced through chemical or optogenetical stimulation, has been shown to promote Tau secretion, accumulation, aggregation, and cell-to-cell propagation (*74*, *75*). These are key processes in the development and spread of Tau pathology in AD and FTD and may also contribute to the progression of Tau pathology in IgLON5 disease. Susceptibility of neurons to hyperexcitation could, therefore, encode a selective vulnerability of neuronal circuits to Tau pathology. The here revealed hyperactivity-induced Tau pathology in anti-IgLON5 disease emphasizes the importance of considering the modulation of neuronal activity for therapeutic approaches to prevent or lower Tau pathology and toxicity across tauopathies. For the treatment of anti-IgLON5 disease patients, determining the neuromodulatory activity of their plasma AAB pool could help in the choice of treatment, i.e., whether to target neuronal hyperexcitation, neuroinflammation, Tau phosphorylation, and aggregation, or all. For example, our data indicate that the effects of α-IgLON5 AABs on neuronal activity and Tau depend on AAB binding strength, AAB concentration, and duration of exposure. These factors differ between patients and may contribute to variations in brain functional impairment and Tau pathology progression between anti-IgLON5 patients (*8*). Further, whether neuronal hyperactivity plays a role for Tau pathology in other immune-related secondary tauopathies, e.g., in subacute sclerosing panencephalitis (*76*) (measles late reaction) and head-nodding syndrome (*77*) (involving DJ-1 antibodies), should be tested as well.

In mice with cerebroventricular infusion of α-IgLON5 AABs, Tau phosphorylation occurred in hippocampal projections (i.e., MFs between DG and hippocampal CA3, projections onto ventral CA1, and commissural fibers in CC). A similar projection selectivity of phosphorylated or misfolded Tau accumulation in MFs has, for example, been observed in mice expressing acetylation-mimetic or pro-aggregant Lys^280^-deletion Tau (*78*, *79*), where it was associated with deficits in synaptic plasticity in this circuit. Whether α-IgLON5 AAB–treated mice show Tau phosphorylation in neurons of locus coeruleus or entorhinal cortex, two other regions primary affected by Tau pathology in AD (*80*–*82*), remains to be tested. The α-IgLON5 AAB–induced accumulation of phosphorylated Tau in axonal projections, and not (yet) neuronal cell bodies, is reminiscent of earliest pathological alterations found in other tauopathies, like AD and FTD, where changes in Tau (i.e., phosphorylation and somatodendritic missorting) spatially associate with progressive decay in synaptic homeostasis (*34*, *83*–*85*) and neuroinflammation and, ultimately, lead to neurodegeneration (*8*, *9*) (Supplementary Text). A previous pilot study suggested that the cerebroventricular infusion of IgGs from an IgLON5 disease patient into mice expressing human wild-type Tau [hTau line; (*86*)] increased the extend of Tau phosphorylation (AT8) in hippocampal and brain stem neuronal cell bodies (*54*); neuronal loss was not reported in this study. It remains to be tested whether administration of α-IgLON5 AABs in models developing Tau pathology (e.g., transgenic mice expressing FTD-mutant Tau) would promote Tau aggregation, supportive of our in vitro data in TauP301L/S320F-expressing neurons, and induce neurotoxicity. In addition, α-IgLON5 AABs triggered microglia activation, neuroinflammatory gene expression signatures (TNFα and IL-6 pathways) commonly observed in Tau and neurodegeneration models, and a pronounced up-regulation of Ig gene expression that is typically not reported in tauopathy brains or models. The latter may indicate B cell infiltration, e.g., as a result of α-IgLON5 AAB–induced IL-6 up-regulation (*87*), or inflammation-related microglial Ig expression (*88*). Mild B cell infiltration has been reported in IgLON5 autopsy brains (*22*). Whether Ig gene up-regulation occurs in IgLON5 disease patient brains is not known. The IgG subclass composition of α-IgLON5 AAB in the brain may play an important role for the extend and kind of neuroinflammation induced (Supplementary Text). As in other tauopathies, however, it remains uncertain whether α-IgLON5 AAB–induced neuroinflammation is mediated by Tau changes and to what extent neuroinflammation contributes to Tau pathology.

α-IgLON5 AAB–induced Tau phosphorylation in MF and CC axonal projections appeared to be epitope selective on epitope pS396/pS404. This is in line with previous reports that identified (pre)synaptic Tau is phosphorylated at pS396/pS404 (*89*, *90*), an epitope targeted by multiple kinases, including GSK3β, CDK5, and ERK (*91*, *92*). Previous studies indicated that Tau/GSK3β interactions are important for synaptic defects in neurodegenerative diseases (*93*, *94*) and that GSK3β is especially important for synaptic Tau phosphorylation at pS396/pS404 (*95*). In vitro, GSK3β phosphorylates Tau directly with a preference at the pS396/pS404 epitope, thereby promoting its aggregation into AD-like fibrils (*92*). In addition, phosphorylation of Tau can be primed by cAMP-dependent PKA, leading to strong phosphorylation involving additional Tau epitopes (*91*). Whether α-IgLON5 AAB–induced MF Tau phosphorylation at pS396/pS404 relates to specific kinases, e.g., GSK3b, needs to be further investigated. Notably, in IgLON5 patient brains, synaptic and axonal Tau phosphorylation has not yet been assessed. However, a recent study on IgLON5 disease brain stem Tau pathology suggested a disease stage dependent occurrence of Tau phosphorylation sites in neuronal somata (*96*), which indicated sequential occurrence of AT8 > pT231 > pS396 and additional phospho-epitopes. In AD hippocampus, Tau epitopes also seem to occur sequentially yet different from IgLON5 patient brain stem (*97*–*99*). It is important to note though that all these studies characterize phospho-Tau accumulating in neuronal cell bodies, and not in axonal projections as observed in α-IgLON5 AAB–treated mice in our data.

Clinically, anti-IgLON5 patients generally present with complex sleep and breathing disturbances, and neurophysiological assessment relies primarily on video polysomnography. Epileptiform electroencephalogram (EEG) activity has been reported in a subset of anti-IgLON5 patients (*100*), and EEG abnormalities are variable and often absent in routine recordings (*3*, *6*). Accordingly, data from a pilot study with intracerebroventricular infusion of α-IgLON5 AABs in wild-type mice did not also reveal consistent EEG or behavioral (e.g., sleep, breathing, and licking behavior) abnormalities (*54*). Therefore, our findings on α-IgLON5 AAB–induced hyperexcitability in dissociated neuronal cultures may not directly translate into a globally hyperactive brain state. Instead, such changes at the cellular level may alter how neurons integrate synaptic inputs, affecting synaptic plasticity and network activity without producing overt population-level hyperactivity. Additionally, transient changes in local neuronal activity of anti-IgLON5 patients may be missed by single time-point assessments through EEGs, especially early in the disease when mostly brain stem and hypothalamus, but not cortex or hippocampus, are affected. In animal models, brain region–specific continuous or longitudinal measurements of neuronal activity using single- or multielectrodes or Ca^2+^ imaging will, therefore, be critical to capture the temporal dynamics of anti-IgLON5 AAB–induced cellular hyperexcitability and circuits dysfunction.

## MATERIALS AND METHODS

### Ethics

All analyses of patient material were approved by the Charité - Universitätsmedizin Berlin Ethics Board (no. EA1/258/18). Primary mouse neuron preparations and experiments involving adult animals (C57/B6, male) were carried out according to the guidelines stated in the directive 2010/63/EU of the European Parliament on the protection of animals used for scientific purposes and were approved by local authorities in Berlin and the animal welfare committee of the Charité Universitätsmedizin Berlin, Germany.

### IgLON5 patients

Anti-IgLON5 disease patients, whose α-IgLON5 ABB and pCtrl AB pools were isolated in this study, were patients in the neurology clinics of the Charité University Medicine Berlin (patients: IgLON5#2,3,4) and of the Otto-von-Guericke-University Magdeburg (patient: IgLON5#1) (Table 1). Phosphorylated tau 217 (pTau217) in serum of patients IgLON5#2,3,4 and in plasmapherisate of patient IgLON5#1 was measured using the Simoa pTau217 Advantage PLUS assay (Quanterix, Billerica, MA, USA; kit lot 504407) at the DZNE Bonn. The intraplate coefficient of variation was <5.1% based on two control levels run in duplicates at the beginning and end of the plate.

**Table 1.** Anti-IgLON5 disease patient information. MP, methylprednisolone; PPH, plasmapheresis; RTX, rituximab; IA, immunoadsorption; IVIG, intravenous immunoglobulins; *, pTau217 in plasmapherisate of this patient; M, male; F, female; Y, years; N.A., not applicable.

Patient#	Sex (M/F)	Age (Y)	Immunotherapy	Effect?	AAB titer	CSF NFL	Serum pTau217 (pg/ml)	Clinical symptoms
IgLON5#1	M	59	MP and PPH	N.A.	1:1000 (serum) and 1:100 (CSF)	N.A.	0.0722*	Sleep apnea, myoclonic jerks, dysarthria, and gait disorder
IgLON5#2	M	59	PPH, RTX, and Bortezomib	No	1:1000 (serum) and 1:100 (CSF)	841	0.0193	Gait instability, bulbar symptoms, sleep disturbance, fasciculations, myoclonic jerks, myorhythmia, and hyperreflexia
IgLON5#3	F	35	IA and RTX	No	1:3200 (serum) and 1:32 (CSF)	559	N.A.	Sleep disturbance and fasciculations
IgLON5#4	F	76	MP and PPH IVIG	No	1:1000 (serum) and 1:10 (CSF)	1127	0.0246	Bulbar syndromes, sleep disturbance, respiratory insufficiency, and cognitive dysfunction

### α-IgLON5 AAB detection in patient CSF and serum

Specificity of the purified α-IgLON5 AABs was confirmed by indirect IF performed on HEK293 cells transfected with commercially available IgLON5 expression vector (EUROIMMUN, no. 1151-1005-50) and cells with control transfection. α-IgLON5 AAB binding on cells was confirmed at dilutions of 1:100 for CSF and 1:1000 for serum.

### Purification of patient-derived α-IgLON5 AABs

Recombinant human IgLON5-Fc chimera (R&D Systems) was bound to a 1-ml HiTrap NHS-activated HP column (GE Healthcare) according to the manufacturer’s guide. For antibody isolation, eluates of plasmapheresis were loaded overnight onto the column. Then, the column was washed with 15 ml of 20 mM tris (pH 7.5) and 15 ml of 0.5 M NaCl in 20 mM tris (pH 7.5). α-IgLON5 AABs were eluted with 100 mM glycine (pH 2.2) with subsequent neutralization using 1 M tris (pH 8.8). Last, the samples were rebuffered into PBS using Vivaspin 15R ultrafiltration spin columns (Sartorius). For detection of specificity, recombinant IgLON5 or LG1 protein was spotted onto a nitrocellulose membrane (GE Healthcare), membranes were blocked with 1% bovine serum albumin (BSA) in PBS, incubated with α-IgLON5 AABs overnight at 4°C, washed, and incubated with HRP-coupled antihuman IgG. For the isolation of control serum IgGs (pCtrl), 750 μl of serum from healthy controls was mixed with 450 μl of protein A/G agarose (Santa Cruz Biotechnology). IgG fractions were isolated according to manufacturer’s instructions. Purity was checked by Coomassie staining. Protein concentration was determined using a bicinchoninic acid (BCA) protein assay kit (Thermo Fisher Scientific). The nonreactive human monoclonal control AAB (mCtrl; mGO53) was purchased from InVivo BioTech.

### IgG subclass composition of patient-derived AABs

For detection of IgG subclasses, 2 μg of patient-derived α-IgLON5 AABs #1 and pCtrl were dotted on a nitrocellulose membrane (GE Healthcare). Membranes were probed with mouse antihuman IgG1-POD, antihuman IgG2-POD, antihuman IgG3-POD, and IgG4-POD ab (Thermo Fisher Scientific) and sheep antihuman IgG (Seramun). For quantitative determination of subclass distribution across all four isolated α-IgLON5 antibody samples and pCtrl, the Human IgG Subclass ELISA Kit (Thermo Fisher Scientific) was used according to the manufacturer’s protocol.

### Relative binding strength quantification using flow cytometry

Binding strength was determined following previously published protocols (*101*). In brief, HEK293T cells were transfected with cDNA plasmids coding for full human IgLON5 protein that coexpressed a myc-tag for transfection efficiency control. Three days posttransfection, cells were harvested and stained with serial dilutions of AABs and c-myc AABs. From live cells with top 30% protein expression (evaluated by c-myc signal), the mean fluorescence intensity (MFI) of Alexa Fluor 488–coupled goat antihuman IgG (1:500; Life Technologies) was evaluated, and nonlinear regression models [MFI = MFI_max_*IgG conc./(0.5*MFI_max_ + IgG conc.)] under settings for one site-specific binding were generated using GraphPad Prism 8 (GraphPad Software Inc.).

### Tissue reactivity screening

Sagittal mouse brain sections were obtained from unfixed tissue, cut on a cryostat (Leica) at 20-μm thickness, and mounted on glass slides. Sections were rinsed with PBS, then blocked with PBS containing with 2% BSA and 5% normal goat serum (NGS) for 1 hour at room temperature, and incubated with α-IgLON5#1 and pCtrl overnight at 4°C. After three washes with PBS, goat antihuman IgG–Alexa Fluor 488 was applied for 2 hours at room temperature, followed by staining with 4′,6-diamidino-2-phenylindole (DAPI; 1:1000 in PBS) for 5 min. Sections were washed with PBS before mounting with Immo-Mount (Epredia) and imaging using an inverted epifluorescence microscope (Leica SPE).

### Epitope mapping in HEK293T cells expressing IgLON5 constructs

To map the epitopes of patient-derived α-IgLON5#1, we cloned human IgLON5 deletion constructs from full-length Myc-DKK-tagged human IgLON5 plasmid (OriGene, no. 225495) using a Q5 Site-Directed Mutagenesis kit (New England Biolabs). Individual Ig domains of IgLON5 (Ig1, Ig2, and Ig3) or combinations thereof (Ig1 + Ig2, Ig1 + Ig3, and Ig2 + Ig3) were expressed in HEK293T cells for 48 hours. HEK293T cells were seeded on poly-l-lysine (PLL)–coated coverslips and transiently transfected with either full-length IgLON5 or its deletional constructs. After fixation with 4 % PFA for 10 min at room temperature, blocking with 3% NGS in PBS for 1 hour, cells were incubated with α-IgLON5 AABs or pCtrl overnight at 4°C. After washing and incubation with the Alexa Fluor 488–conjugated antihuman IgG for 1 hour at room temperature, binding of α-IgLON5 AABs was evaluated on the basis of epifluorescence microscope (Leica SPE).

### Intrathecal osmotic pump infusion

Eight-week-old male C57Bl/6J wild-type mice were randomized for the different treatment groups by an independent investigator. In total, 75 μg (~3 μg/g body weight) of α-IgLON5#1, IgG from healthy control individual serum (pCtrl), and PBS were delivered continuously into the right lateral ventricle over the course of 14 days, with a flow rate of 0.25 μl/hour. The cohort consisted of α-IgLON5 (*n* = 10), pCtrl (*n* = 9), and PBS (*n* = 9) animals. Antibody cerebroventricular infusion was performed unilaterally using osmotic pumps (model 1002, Alzet, Cupertino, CA), which were loaded 24 hours before surgical implantation. For pump implantation, mice were placed in a stereotaxic frame, and a cannula was inserted into the right ventricle (coordinates: 0.2 mm posterior and ±1.00 mm lateral from bregma, depth of 2.2 mm). The cannula was connected to a pump, which was subcutaneously implanted on the interscapular space of the animals. After surgery, mice were monitored daily to assess symptoms and body weight. Mice were euthanized after 14 days and brain, and serum were harvested.

### Tissue processing

For protein and RNA analysis, brain and spinal cord tissues were promptly dissected on ice, flash frozen by placing on dry ice, and stored at −80°C until further use. For protein extraction, brain and spinal cord tissues were homogenized in ice-cold radioimmunoprecipitation assay (RIPA) buffer (Sigma-Aldrich) containing phosphatase and protease inhibitor cocktail (Thermo Fisher Scientific) using a probe homogenizer. After incubating the homogenates on ice for 20 min, samples were centrifuged at 10,000*g* for 20 min at 4°C. Protein concentration in the supernatant was measured using a BCA protein assay kit (Pierce). Concentration of each sample was adjusted to 2 μg/μl and then mixed with 6× SDS-containing Laemmli buffer (Thermo Fisher Scientific). Subsequently, they were boiled at 95°C for 5 min and stored at −20°C until the Western blot analysis.

For immunohistochemistry (IHC), brains were drop fixed in 4% PFA for 2 days. For cryoprotection, fixed tissues were sequentially transferred to 10, 20, and 30% sucrose in PBS with 0.02% sodium azide at 4°C. Tissue was cut into 30-μm-thick sections using a cryostat (Leica) and serially collected in 50% glycerol in PBS to be stored at −20°C until used for IHC.

### Western blot

To analyze total and p-Tau levels by Western blot, 10 μg of total protein per sample was loaded onto 4 to 12% bis-tris SDS-PAGE (Invitrogen). Following electrophoresis, the separated proteins were transferred to a nitrocellulose membrane. The membrane was blocked with 3% BSA in PBS containing 0.05% of Tween (PBS-T) for 1 hour at room temperature. Subsequently, the membranes were incubated with primary antibodies for total Tau, p-Tau, and loading control, diluted in the blocking solution, overnight at 4°C. The following day, membranes were washed three times for 5 min in PBS-T and then incubated with fluorescent dye–conjugated secondary antibodies, diluted in blocking solution, for 1 hour at room temperature. Membranes were then imaged using a LI-COR imaging system (Odyssey DLx). A full list of antibodies used in Western blot can be found in table S1.

### Histology

For immunodetection, 30-μm-thick PFA-fixed brain sections were washed free-floating in tris-buffered saline (TBS), permeabilized with 0.3% Triton X-100 in TBS for 20 min at room temperature, and washed two times for 5 min in TBS. All incubation steps were performed on an orbital shaker (180 rpm). Sections were blocked in blocking solution (3% NGS in TBS) for 1 hour at room temperature. Sections were incubated with primary antibodies diluted in blocking solution at 4°C overnight. Next day, the sections were washed three times for 10 min in TBS and incubated with Alexa Fluor–conjugated secondary antibody diluted in blocking solution for 2 hours at room temperature. Sections were washed three times for 10 min in TBS and incubated with DAPI (1:1000 in PBS) for 15 min at room temperature. Sections were then mounted onto microscope slides and covered with coverslip using Fluoroshield mounting medium (Sigma-Aldrich). A full list of antibodies used IHC can be found in table S1. Sections were imaged on a wide field fluorescence microscope (Eclipse-Ti, Nikon) using 10× objective with tiling function. Same imaging parameters were used while imaging the sections across different groups. MFI was measured using ImageJ from manually drawn regions of interest (ROIs) in the brain sections (on contralateral site to avoid injection confound). Mean intensity was averaged over three to five brain sections per animal.

Detection of the spread of infused AABs was done in free-floating brain sections with goat antihuman HRP-conjugated secondary antibody in 3% NGS in TBS for 2 hours at room temperature. Sections were washed three times for 5 min in TBS, and the signal was amplified with a CF 594 tyramide signal amplification kit (Thermo Fisher Scientific).

### Evaluation of serum

Levels of Nfl was measured from undiluted serum samples using a commercial ELISA kit (Cloud-Clone) according to the manufacturer’s instructions.

### RNA sequencing

Total RNA was extracted from brain tissue using the miRNeasy Micro Kit (QIAGEN) according to the manufacturer’s protocol. RNA integrity was assessed before bulk RNA-seq, which was performed in paired-end mode using the Illumina NovaSeq 6000 platform. Raw FASTQ files were aligned to the GRCm38 reference genome using STAR aligner with default parameters (*102*). The following analysis steps were performed in R (v4.3.0) (R Core Team, R: A language and environment for statistical computing, R Foundation for Statistical Computing) and R Studio [v1.4.1717; RStudio Team, RStudio: Integrated development for R, RStudio Inc., Boston, MA (2021)]. Genes with fewer than 10 read counts in at least four samples were excluded from the analysis, resulting in a filtered dataset of 20,271 genes for downstream processing. Normalization of the count matrix was computed with R/DESeq2 (v1.40.2) (*103*) and a variance stabilizing transformation applied using the DESeq2 vst function at default settings. Differential expression analysis based on the DESeq2 package was performed adjusting *P* values according to independent hypothesis weighting from the R/IHW package (v1.28.0) (*104*) and applying apeglm shrinkage from the R/apeglm package (v1.22.1) (*105*). Differentially expressed genes (DEGs) were defined on the basis of a fold change threshold of >2 and a *P* value threshold of <0.05. A gene set enrichment analysis was performed with the transformed data as the input using R/fgsea (v1.26.0) (*106*), whereby the GO (*107*) and the Molecular Signature Database (MSigDB) (*108*, *109*) Hallmark gene set were used.

### Preparation of mouse primary neurons

Primary neurons were prepared from hippocampi dissected from postnatal 0 (P0) to P1 wild-type mice of either sex. Hippocampi were dissected in ice-cold Hanks’ balanced salt solution (Merck Millipore) containing 1% penicillin/streptomycin (P/S). Then, hippocampal tissue was digested in enzyme solution containing Dulbecco’s minimum essential medium (DMEM; Thermo Fisher Scientific), 3.3 mM cysteine, 2 mM CaCl_2_, 1 mM EDTA, and papain (20 U/ml; Worthington) at 37°C for 30 min. Papain reaction was inhibited by incubating digested hippocampal tissue in DMEM containing 10% fetal bovine serum (FBS; Thermo Fisher Scientific), 1% P/S, 38 mM BSA, and 95 mM trypsin inhibitor at 37°C for 5 min. Cells then were triturated in complete Neurobasal-A (NBA) medium containing 10% FBS, 2% B-27, 1% GlutaMAX, and 1% P/S and seeded in PLL (0.1 mg/ml)–coated glass bottom μ-slide eight-well imaging dishes (ibidi) at a seeding density of ~30,000 cells/cm^2^ for IF experiments. For calcium (Ca^2+^) imaging, neurons were seeded in 96-well clear-bottom black microplates (Corning) at a seeding density of 125,000 cells/cm^2^ and maintained at 37°C and 5% CO_2_ until used for experiments. After 3 hours of seeding the neurons, the medium was completely changed to FBS and phenol red–free complete NBA medium. Every second day, one-fifth of the medium was replaced by fresh complete NBA medium.

### Differentiation of human neurons

Human neuronal stem cells (hNSCs) were differentiated using an inducible Neurog2 hNSCs through doxycycline induction. hNSCs were received from Berlin Institute of Health Core Unit pluripotent Stem Cells and Organoids, Charité-Berlin. Cells were seeded in laminin 521 (Biolamina)–coated eight-well plates. The composition of the differentiation medium was NBA, Neural Induction Supplement, and Advanced DMEM/F12 (Gibco), supplemented by antibiotic (Gibco) and doxycycline (Sigma-Aldrich). On day 4, the medium change was performed by the medium supplemented with AraC (Sigma-Aldrich) to reduce the number of nonneuronal cells. The differentiation medium was refreshed every day, and the cells were cultivated for 43 days in 37°C and 5% CO_2_.

### Antibody binding curves on the neuronal surface

Primary mouse hippocampal neurons (DIV12) were fixed with 4% PFA for 15 min at room temperature and blocked with 3% NGS in PBS for 1 hour. For surface staining with AABs, the permeabilization step was omitted. Neurons were then incubated overnight at 4°C with increasing concentrations (0.01, 0.1, 1, and 10 μg/ml) of α-IgLON5 AABs or pCtrl diluted in blocking buffer. The next day, cells were washed with PBS and incubated for 2 hours at room temperature with Alexa Fluor 488–conjugated α-human secondary antibodies. After additional PBS washes, neurons were permeabilized with 0.3% Triton X-100 in PBS for 20 min, reblocked, and stained overnight at 4°C with MAP2 primary antibodies. Alexa Fluor 555–conjugated secondary antibodies were applied for 2 hours at room temperature the following day. Nuclear staining was performed using DAPI (1:1000 in PBS, 10 min at room temperature), followed by a final PBS wash. Neurons were imaged using a spinning disk confocal microscope (Nikon CSU-X) equipped with a 10× objective lens. Quantitative analysis of antibody binding was done by measuring the MFI within dendritic regions. Dendrites were identified on the basis of MAP2-positive staining and delineated using CellProfiler software. The MFI was measured for each treatment concentration using ImageJ software. Concentration-dependent binding curves were then generated.

### Live staining (surface antigen binding)

Primary mouse hippocampal neurons (DIV12) were treated with α-IgLON5 AABs or control antibodies (1 μg/ml) and kept at 37°C and 5% CO_2_ for 60 min. Then, neurons were washed with PBS before fixation with 4% PFA in PBS for 15 min and then washed with TBS for 10 min at room temperature. After fixation, neurons were blocked with 3% NGS for 1 hour at room temperature and incubated with antihuman 488 secondary antibodies for 2 hours at room temperature. After three times 10 min PBS washes, nuclei were stained with DAPI (1:1000 in PBS) for 10 min. Dishes were imaged with laser scanning confocal microscope (Nikon, A1Rsi+) with a 60× oil objective.

### Colocalization with synaptic markers

After the surface antigen labeling as described above, neurons were permeabilized with PBS containing 0.3% Triton X-100 for 20 min at room temperature. After two consecutive washing steps with excess PBS, neurons were blocked again with 3% NGS for 1 hour. Neurons then were incubated with primary antibodies, diluted in blocking solution, for microtubule (MAP2) and presynaptic (synapsin-1) or postsynaptic (PSD95) markers overnight at 4°C. Secondary antibody and DAPI stainings were performed as described above. Dishes were imaged with a laser scanning confocal microscope (Nikon, A1Rsi+) with a 60× oil objective using the *z*-stack function (*z*-step, 0.5 μm). Same imaging settings were used between each biological replicate (*n* = 3) to allow the comparison for colocalization analysis, which was performed using standard procedures and custom-made macros in ImageJ.

### IgLON5 KD in Neuro2a cells and primary mouse neurons

To knock down mouse IgLON5, pAAV-U6-shRNA vectors encoding either IgLON5-shRNA or scramble-shRNA, both expressing a BFP marker, were obtained from VectorBuilder. Knockdown efficiency was first validated in Neuro2a mouse neuroblastoma cells (American Type Culture Collection, CCL-131). Cells were cultured to ~60% confluence in DMEM supplemented with 10% FBS, 1% P/S, 2 mM l-glutamine, and 1% NEAA and transfected using Lipofectamine 2000 per the manufacturer’s protocol. After 24 hours, cells were fixed with 4% PFA, and surface IgLON5 staining was performed using α-IgLON5#1. Cell membranes were labeled with CellBrite-Red (Biotium). Imaging was conducted on a spinning disk confocal microscope (Nikon CSU-X, 40× objective). Following validation, AAV2/9 viral particles were produced by the Viral Core Facility at Charité [IgLON5-shRNA: 8.26 × 10^12^ vector genomes (VGs)/ml; scramble-shRNA: 9.86 × 10^12^ VGs/ml]. At DIV5, primary mouse hippocampal neurons were transduced with serial dilutions (0.001 to 0.5 μl) of IgLON5-shRNA or scramble-shRNA AAVs. At DIV12, cells were fixed and stained for surface IgLON5 using α-IgLON5#1, followed by MAP2 staining for dendritic visualization. Imaging was performed on a spinning disk confocal microscope (Nikon CSU-X, 10× objective). Quantification of α-IgLON5 AAB binding was done by measuring MFI in dendritic regions of IgLON5-shRNA and control neurons.

### Tau missorting analysis in neurons

Primary neurons were treated with different concentrations of α-IgLON5 AABs or control antibodies for either 2 days or 5 days. For Ca^2+^ depletion experiments, neurons were treated with 10 μM cell-permeable Ca^2+^ chelator, EGTA-AM (AAT Bioquest), 20 min before the antibody treatment. After the treatments, neurons were fixed at DIV9 to DIV12 with 4% PFA for 15 min and immunolabeled for total Tau and MAP2 by following the IF protocol described above. Antibody specifications are listed in table S1. Neurons were imaged with a spinning disc confocal microscope (Nikon, CSU-X), using a 40× oil objective. Identical imaging settings were used for all conditions. ROIs were manually defined for the soma and nucleus on the basis of signals from the MAP2 and DAPI channels, respectively. Mislocalization of Tau into the somatodendritic compartment was quantified by calculating MFI from the Tau channel using the following equation

$$\text{MFI}=\frac{{\text{RawIntDen}}_{\text{soma}}-{\text{RawIntDen}}_{\text{nucleus}}}{{\text{Area}}_{\text{soma}}-{\text{Area}}_{\text{nucleus}}}$$

### FTD-Tau aggregation analysis in neurons

Primary neurons (DIV5) were transduced with AAV2/9 serotype viral particles encoding green fluorescent protein (GFP)–tagged human full-length Tau (2N4R isoform) carrying two FTD-associated mutations (ΔK280, P301L, and P301L/S320F) under human synapsin 1 (hSyn1) or CAG promoter. At DIV12, neurons were treated with either pCtrl or α-IgLON5 AABs (1 μg/ml) for 2 days, fixed on DIV14, and immunolabeled for MAP2 and DAPI. GFP-TauΔK280– and GFP-TauP301L–expressing neurons were treated with AABs for 7 days. Tau aggregates were manually counted and the percentage of neurons with tangle-like Tau aggregates calculated as # tangles/# DAPI + nuclei.

### Cytotoxicity assay (LDH)

To assess cytotoxicity, neuronal culture supernatants were collected, centrifuged at 1000*g* for 5 min, and analyzed using the CyQUANT lactose dehydrogenase (LDH) assay (Thermo Fisher Scientific) per the manufacturer’s instructions. Absorbance was measured at λ = 490 nm in Tecan Infinite M Plex plate reader.

### Ca^2+^ imaging in cultured neurons

For Ca^2+^ imaging in cultured hippocampal neurons, AAV2/9 serotype was used for neuron-specific expression of GCamp6f under the hSyn promoter. AAVs expressing hSyn:mRuby2-P2A-GCamp6f were produced by the Viral Core Facility of Charité Universitätsmedizin, Berlin (catalog number BA-026e). AAVs (0.16 μl of 1.04 × 10^13^ VGs/ml) were added to hippocampal neurons cultured in 96-well plates at DIV5. Ca^2+^ recordings were performed at DIV13 to DIV15. Additionally, immunostaining for c-FOS, an immediate early gene marker of neuronal activation, was performed in treated neurons to confirm AAB-induced hyperactivity.

#### Ca^2+^ transient recordings

Neurons in 96-well plates expressing GCamp6f were imaged using a wide field fluorescence microscope (Eclipse-Ti, Nikon) with a 10× objective. Imaging dishes were placed in the live-cell imaging chamber of the microscope to maintain an environment of 37°C, 5% CO_2_, and ~95% humidity. α-IgLON5 AABs or control antibodies were added to the wells by direct application. Bicuculline (30 μM for 5 min) and TTX (0.5 μM for 10 min) were included in the recordings as controls. Ca^2+^ imaging was done in the green channel– and mRuby-expressing neurons were visualized in the red channel. Ca^2+^ transients were recorded for 180 s at ~8 Hz. Two to three wells in three independent cultures were analyzed per condition.

#### Analysis

ROIs were created for each soma in the red channel using a custom-written script in CellProfiler. Created ROIs were placed over each frame of the video recorded in the green channel, and mean intensity over time was measured using ImageJ. The .csv files containing MFI from each image of the time-lapse videos were processed using the FluoroSNAP application for Δ*F*/*F*0 conversion and spike detection (*110*). In short, .csv files were uploaded to FluoroSNAP, and the function “Convert raw fluorescence data to Δ*F*/*F*0” was used to compute baseline fluorescence by taking the average of the 50th percentile of the signal across a 60-s time window. Ca^2+^ transients from individual ROIs were detected with a template-based approach. This method identifies events by comparing a moving window of the Ca^2+^ signal to a predefined library of Ca^2+^ waveform templates. Events were detected with a similarity threshold of 0.75 and a minimum amplitude of 0.01. Hyperactivity criteria were calculated for each time point independently by taking the means ± SD of the pCtrl group as a reference value for normal activity: Spike frequencies larger than [means + 2*SD] of pCtrl were considered as hyperactivity (*111*, *112*).

### Electrophysiology in primary autaptic cultures

Isolated primary neurons on micro islands of glial cells (“autaptic cultures”) were prepared from wild-type mice as recently described (*113*), with slight modifications. Briefly, 300-μm spots of a growth-permissive substrate mix of collagen (0.7 mg/ml) and poly-d-lysine (0.1 mg/ml) were printed on glass coverslips coated with agarose. Astrocytes were seeded onto these coverslips in DMEM (Thermo Fisher Scientific), supplemented with 10% fetal calf serum and 0.2% P/S (Invitrogen). After formation of glia microislands, DMEM was replaced with NBA supplemented with 2% B27 and 0.2% P/S. Hippocampal neurons prepared from P0 mice were added at a density of 370 cells/cm^2^. Before electrophysiological recordings, autaptic cultures were treated with antibodies at a final concentration of 0.1 μg/ml for 3 days.

#### Electrophysiological recordings

Neurons were recorded at DIV13 to DIV17 at room temperature on an IX73 inverted microscope (Olympus) using a MultiClamp 700B amplifier under the control of a Digidata 1550 AD board and Clampex 10 software (all Molecular Devices). Data were acquired at 10 kHz and filtered at 3 kHz, and series resistance was compensated at 70%. The extracellular solution contained 140 mM NaCl, 2.4 mM KCl, 10 mM Hepes, 10 mM glucose, 2 mM CaCl_2_, and 4 mM MgCl_2_ (pH adjusted to 7.3 with NaOH, 300 mosmol). The intracellular solution contained 136 mM KCl, 17.8 mM Hepes, 1 mM EGTA, 4.6 mM MgCl_2_, 4 mM Na_2_ATP, 0.3 mM NaGTP, 12 mM disodium phosphocreatine, and creatine phosphokinase (50 U/ml), pH adjusted to 7.3 with KOH, 300 mosmol. Autaptic neurons were recorded in whole-cell voltage clamp mode using thick-walled borosilicate pipettes with a tip resistance of 3 to 4 MΩ. Membrane potential was set to −70 mV. Paired EPSCs were evoked every 5 s by triggering two unclamped action potentials with 40-ms interstimulus interval using 1-ms depolarizations of the soma to 0 mV. The readily-releasable pool of synaptic vesicles was determined by application of a hypertonic 500 mM sucrose solution for 10 s. Electrophysiological recordings were analyzed using AxoGraph. The vesicular release probability was calculated as the ration of the charge of the sucrose evoked response by the average charge of six EPSCs before the sucrose application. The paired-pulse ratio was calculated as the ratio from the second and first EPSC amplitude.

### Antibody internalization experiments

α-IgLON5 AABs and pCtrl were conjugated with endosomal-pH sensitive red-fluorescent dye (pHrodo iFL Red STP ester, Thermo Fisher Scientific) following the manufacturer’s instructions. pHrodo-conjugated antibodies were applied at 5 μg/ml to primary mouse hippocampal neurons (DIV12) for 2 days at 37°C. Images were captured on a spinning disc confocal microscope (Nikon, SoRa CSU-W1).

### Cell surface clustering of α-IgLON5 AABs

Live primary mouse neurons (DIV12) were treated with α-IgLON5#1 or pCtrl (1 μg/ml) or left untreated (NT) and incubated at 37°C and 5% CO_2_ for 60 min. After incubation, cells were washed with PBS, fixed with 4% PFA, and surface stained using α-IgLON5 AABs as described in the “Antibody binding curves on the neuronal surface” section. MAP2 staining was used to visualize dendrites. Imaging was performed on a Nikon A1Rsi+ laser scanning confocal microscope using a 60× oil objective with *z*-stack (0.5-μm steps), applying consistent settings across biological replicates. IgLON5 cluster density, size, and radius were quantified using a custom CellProfiler pipeline.

### Fab fragment preparation and cell treatments

Fab fragments were generated from α-IgLON5#1 using a commercially available Fab preparation kit (Thermo Fisher Scientific) following the manufacturer’s protocol. Briefly, IgG was digested with papain to cleave the Fc region and isolate antigen-binding Fab fragments. The resulting fragments were separated via SDS-PAGE to confirm digestion efficiency. For all Fab assays, neurons were treated with equimolecular concentrations of α-IgLON5#1, α-IgLON5#1 fab fragment, or pCtrl.

### Antibody-mediated cell surface proximity biotinylation

Primary mouse neurons (DIV12) were treated with α-IgLON5#1 or pCtrl (1 μg/ml) for 60 min at 37°C. After washing with PBS, cells were fixed with 4% PFA for 10 min at room temperature, quenched with 100 mM glycine (10 min), and treated with 0.5% H_2_O_2_s (10 min) to block endogenous peroxidase. Following PBS washes, cells were blocked with 5% biotin-free BSA for 45 min at room temperature and then incubated with HRP-conjugated α-human secondary antibodies for 1 hour. Surface biotinylation was performed using 250 μM biotin-tyramide (Iris Biotech) and 50 μM H_2_O_2_ for 1 min at t room temperature, followed by quenching with Trolox and sodium l-ascorbate. Cells were washed and prepared for imaging.

#### IF assessment of biotinylation

Cells were stained with streptavidin–Alexa Fluor 555 to detect biotinylated proteins and Alexa Fluor 488–conjugated α-human antibodies to visualize bound α-IgLON5 AABs. Nuclei were counterstained with DAPI. Imaging was conducted on a spinning disk confocal microscope (Nikon CSU-X, 40× oil objective), and colocalization analysis was performed using intensity profiling in ImageJ.

#### Streptavidin pulldown and MS

Neurons were seeded into six-well plates (500,000 cells per well). After surface biotinylation, cells were scraped with PBS, with three wells (~1.5 million cells) pooled per condition. Pooled cells were pelleted by centrifugation at 8000*g* for 5 min at room temperature to remove supernatant. Cell pellet was lysed in ice-cold RIPA buffer (with 0.5% SDS and protease/phosphatase inhibitors). Lysates were sonicated, incubated on ice for 20 min, and boiled (99°C, 1 hour) for decrosslinking. Soluble proteins were collected by centrifugation (14,000*g*, 20 min, 4°C), and protein concentration was determined using a BCA assay (Thermo Fisher Scientific). For pulldown, streptavidin magnetic beads (BioLabs) were equilibrated in RIPA buffer. For each pulldown, 180 μg of protein lysate was incubated with beads overnight at 4°C. Next day, beads were washed with ice-cold RIPA buffer, and biotinylated proteins were separated from the beads through competitive elution by adding 10 mM biotin-tyramide to the bead slurry and by boiling at 95°C for 10 min. The eluted proteins were stored at −80°C for subsequent MS analysis. In addition to the pulldown samples, 30 μg of total protein lysates from α-IgLON5#1 or pCtrl-treated neurons were included in the MS analysis to ensure that the protein composition in the input samples was comparable across treatment conditions.

#### MS sample preparation and analysis

Pulldown (~110 μl) and total lysate (~50 μl) samples were thawed on ice and treated with 0.2 and 0.5 μl of nuclease, respectively. Samples were diluted 1:1 with 50 mM ammonium bicarbonate (ABC). SP3-beads (Sera-Mag SpeedBeads) were prepared as described before (*114*), and 10 μl of beads per pulldown and 15 μl of beads per lysates samples were added. Acetonitrile was added to a final concentration of 70% (v/v), samples were incubated for 30 min at 24°C with shaking (1000 rpm), and supernatants were discarded. Beads were resuspended in 20 μl of 50 mM ABC and reduced with 3 μl of 200 mM DTT at 45°C for 20 min (1200 rpm). After cooling to room temperature, 7 μl of 400 mM iodoacetamide was added, and the samples were incubated at 24°C with shaking at 1200 rpm for 30 min, followed by addition of 3 μl of 200 mM DTT. Freshly prepared 10 μl of SP3-beads were added to the sample still containing previous beads, and ACN was added to a final concentration of 70% (v/v). After incubation for 30 min shaking at 24°C, the beads were washed four times with 80% ethanol, and the supernatant was discarded. For digestion, 20 μl of digestion mix [trypsin (0.03 μg/μl), Lys-C (0.015 μg/μl), and 50 mM ABC] was added per 20 μg of protein. The samples were gently spun and incubated overnight at 37°C. Supernatants were subsequently filtered thorough preequilibrated [0.1% formic acid (FA)] Spin-X 0.22-μm filters and collected in a fresh tube. The remaining beads were resuspended in 20 μl of 0.1% FA and sonicated twice for 30 s in a water bath, and eluates were filtered through Spin-X 0.22-μm filters. Both eluates were combined and dried by vacuum centrifugation. Dried peptides were reconstituted in 13 μl of 0.1% FA. In total, 6 μl of pulldowns and 350 ng of peptides per lysate were injected via a nanoElute nanoHPLC system (Bruker, Germany) coupled to a timsTOF Pro mass spectrometer (Bruker, Germany) with a CaptiveSpray ion source (Bruker, Germany). Samples were separated on an in-house packed C18 analytical column (15 cm by 75 μm inner diameter, ReproSil-Pur 120 C18-AQ, 1.9 μm, Dr. Maisch GmbH) using a gradient of water and ACN (B) at 300 nl/min containing 0.1% FA (0 min, 2% B; 2 min, 5% B; 62 min, 24% B; 72 min, 35% B; and 75 min, 60% B) at a column temperature of 50°C. Spectra were acquired with Data Independent Acquisition Parallel Accumulation–Serial Fragmentation (diaPASEF). Ion accumulation and separation using trapped ion mobility spectrometry (TIMS) was set to a ramp time of 100 ms. One scan cycle consisted of a TIMS full MS scan and 26 windows with a width of 27 mass/charge ratio (*m*/*z*) covering an *m*/*z* range of 350 to 1002 *m*/*z*. Two windows were recorded per PASEF scan, resulting in a cycle time of 1.4 s.

#### MS data processing

MS data were analyzed with DIA-NN version 1.8.2 (*115*). A library free search was performed against a mouse FASTA database including common contaminants. Methionine oxidation and acetylation of protein N termini were set as variable modifications, and carbamidomethylation of cysteine residues was set as fixed modification. The match between runs-option was enabled while data normalization was disabled. Charge states from two to four with an *m*/*z* range of 300 to 1400 were considered. Trypsin with up to two missed cleavages was set as digestion condition. Mass accuracy and ion mobility settings were set to automatic. Statistical data processing was performed with the freeware tool Perseus version 2.0.11 (*116*).

### Statistical analysis

All data plotting and statistical analyses were performed in GraphPad Prism 10. Comparisons between two groups were performed using the unpaired *t* tests, while one-way analysis of variance (ANOVA) followed by Tukey’s test used for multiple group comparisons, as specified in the figure legends. Statistical significance was denoted as follows: *P < 0.05, **P < 0.01, ***P < 0.001, ****P < 0.0001.

### Data and code

RNA-seq data generated in this study are available in the Gene Expression Omnibus (GEO) at www.ncbi.nlm.nih.gov/geo/query/acc.cgi?acc=GSE298951, under the accession number GSE298951. Lists of DEG can be found in data S1. All original code to reproduce key steps of the RNA-seq analysis has been deposited at GitLab (https://gitlab.dzne.de/ag-ulas/iglon5-autoimmune-antibody-influence-on-neurons) and Zenodo (https://doi.org/10.5281/zenodo.19454006).

The proteomics data have been deposited to the ProteomeXchange Consortium via the PRIDE partner repository with the dataset identifier PXD066225. Lists of differentially enriched proteins can be found in data S3.
